# A gas-liquid biphasic nanocleaner for breaking the self-amplified vicious cycle of barrier disruption-inflammation in inflammatory bowel disease

**DOI:** 10.1186/s12951-026-04581-1

**Published:** 2026-05-24

**Authors:** Haibin Wu, Jixiang Zhang, Xinqing Huang, Bo Zhang, Yuting Xie, Qingyu Li, Li Yang, Weiqi Wu, Ziying Zheng, Ziyan Huang, Fanru Gao, Zhongqing Cai, Jiahe Chen, Guohuan Zeng, Daishun Ling, Guang Liang, Qian Chen

**Affiliations:** 1 School of Pharmaceutical Sciences, Hangzhou Medical College, Hangzhou, 311399 Zhejiang China; 2 Department of Pharmacy and Institute of Inflammation, Affiliated People’s Hospital, Zhejiang Provincial People’s Hospital, Hangzhou Medical College, Hangzhou, 310014 Zhejiang China; 3 Frontiers Science Center for Transformative Molecules, School of Chemistry and Chemical Engineering, School of Biomedical Engineering, National Center for Translational Medicine, Shanghai Advanced Research Institute, Shanghai Jiao Tong University, Shanghai, 200240 China; 4 State Key Laboratory of Chinese Medicine Modernization, Innovation Center of Yangtze River Delta, Zhejiang University, Jiaxing, 314102 Zhejiang China

**Keywords:** Gas-liquid biphasic nanocleaner, Self-amplified vicious cycle, Multiphase pathogenic factors, Inflammatory bowel disease

## Abstract

Inflammatory bowel disease (IBD) is a chronic relapsing disorder primarily driven by the self-amplified vicious cycle of colonic barrier disruption-inflammation (VCBI). Especially, these pathogenic mediators of the VCBI often exist in different phases, such as ROS (inflammatory mediators) in the liquid phase and H_2_S (colonic barrier disruptors) in the gas phase, poses a formidable challenge to their concurrent elimination. Herein, a gas-liquid biphasic nanocleaner (GLBN) is developed for breaking the self-amplified VCBI in IBD by concurrently scavenging the pathogenic factors derived from both liquid and gaseous phases. Benefiting from its high specific surface area, superior intrinsic activity, and ultrasmall size, GLBN provides abundant adsorption and active sites, thereby exhibiting highly efficient H_2_S-capturing capability with a scavenging efficiency of 88.6% and attenuating H_2_S-mediated colonic barrier dysfunction. Meanwhile, owing to its highly efficient ROS-scavenging capacity, with respective scavenging efficiencies of 84.3% for H_2_O_2_ and 82.7% for •O_2_^−^, GLBN effectively suppresses inflammation and promotes macrophage repolarization from the M1 toward the M2 phenotype. Moreover, density function Sal theory (DFT) calculations attribute the biphasic scavenging capabilities to heterogeneous facets and metal-sulfur bonding. Consequently, highly efficient therapy of IBD is achieved in a mouse model. Our study marks a critical breakthrough in the therapeutic application of biphasic scavenging nanozymes for inflammatory diseases triggered by multiphase pathogenic factors.

**Graphical Abstract:**

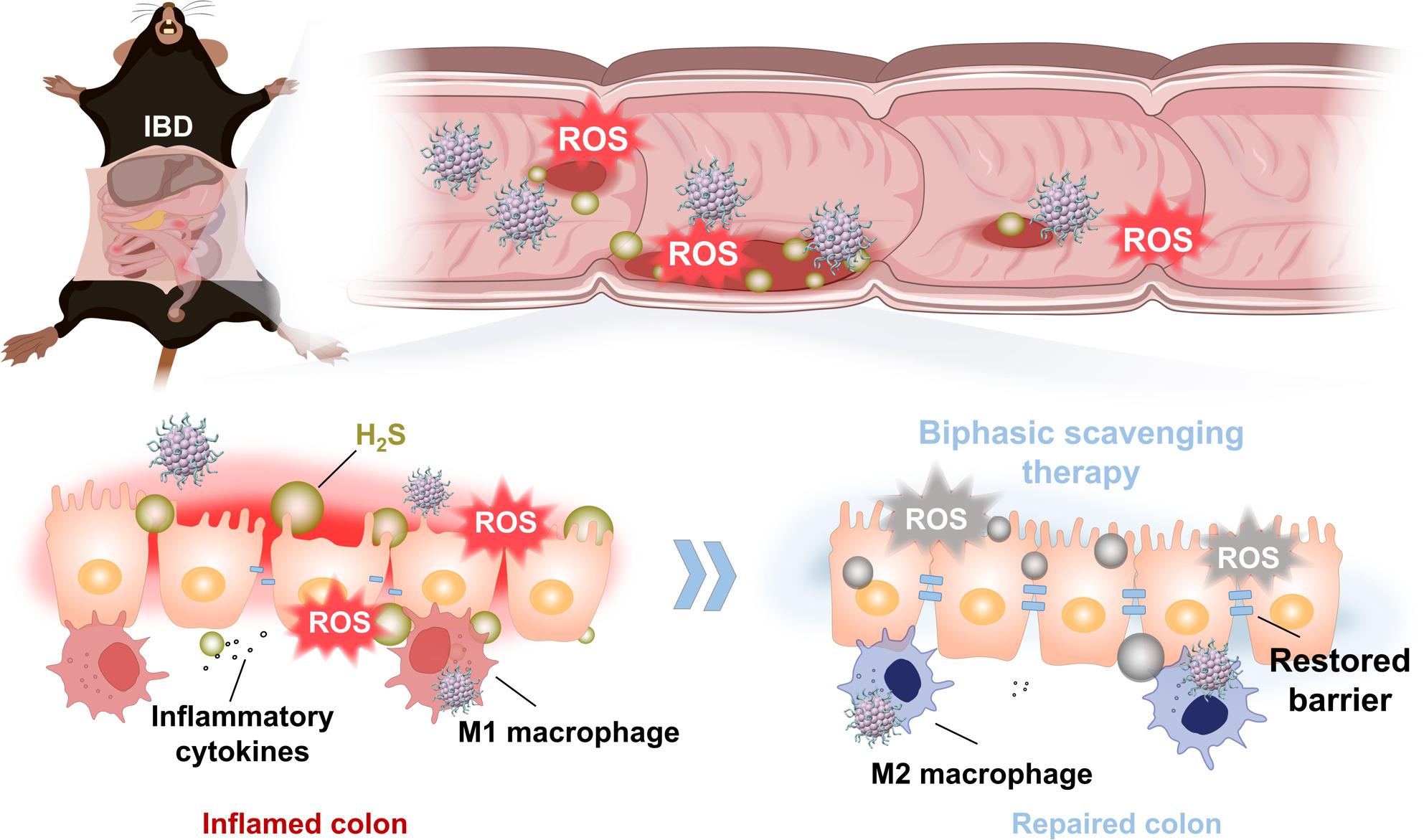

**Supplementary Information:**

The online version contains supplementary material available at 10.1186/s12951-026-04581-1.

## Introduction

Inflammatory bowel disease (IBD) is a chronic, relapsing disorder of the gastrointestinal tract characterized by intractable mucosal inflammation [1, 2], affecting millions globally and imposing a significant healthcare burden [1, 3, 4]. Current therapeutics mainly target single mediators [5–8], while the unresolved pathogenic factors inevitably lead to disease exacerbation and drug resistance. For example, a group of drugs called tumor necrosis factor (TNF) inhibitors (anti-TNF-α agents) have become a significant advance in the management of IBD, which could effectively promote the endoscopic remission and suppress disease progression [9–11]. Nevertheless, approximately 30% of patients are primary non-responders of these anti-TNF-α agents [12]. Among those who do respond initially, the rate of secondary loss of response is considerable, reaching up to nearly 50% within the first year [9, 13]. Therefore, further effective treatment requires thorough understanding of the multifactorial pathological mechanisms that drive the progression of IBD. Recently, a growing number of studies indicate that the pathophysiology of IBD is driven primarily by a self-amplified vicious cycle of colonic barrier disruption-inflammation (VCBI) [14–17]. In this cycle, initial damage to the intestinal epithelial barrier facilitates the translocation of luminal contents, triggering robust immune responses; the resulting inflammation, in turn, exacerbates barrier integrity loss, perpetuating the disease state [15, 17]. Effective therapeutic strategies must, therefore, be capable of concurrently interrupting this self-amplified destructive VCBI loop.

Current IBD treatments, while providing symptomatic relief, often fail to address the VCBI loop induced by the inflammatory microenvironment, which is characterized by multiple pathogenic mediators existing in different physical phases. Particularly, the colonic inflammatory milieu features high concentrations of: (1) reactive oxygen species (ROS) in the liquid phase, which are the dominant oxidative mediators that drive inflammatory responses [18, 19]; and (2) hydrogen sulfide (H_2_S) in the gaseous phase [17, 20], which presents in excess in colitis and acts as a potent colonic barrier disruptor. Specifically, the accumulated H_2_S can destroy the disulfide bonds of the mucin proteins (MUC-2) in the mucus, which can reduce the thickness of mucus layer and compromise intestinal barrier integrity [21]. Even worse, ROS and H_2_S mutually reinforce each other, creating a more severe challenge to their concurrently scavenging. For instance, elevated level of H_2_S could easily induce mitochondrial dysfunction and catalase (CAT) inactivation [22], thus facilitating ROS production and accumulation. Conversely, elevated level of ROS often leads to increased cystathionine γ-lyase (CSE) expression [23], thereby promoting H_2_S production. Moreover, the disparity in the diffusion kinetics and physical states of these two critical mediators (ROS in liquid vs. H_2_S in gas) adds further difficulty to the challenge [24, 25]. Given this, designing a versatile and facile therapy for concurrently scavenging the pathogenic ROS and H_2_S is highly needed but challenging.

Nanozymes, as biomaterials possessing intrinsic enzyme-like catalytic activity, have attracted considerable attention as promising therapeutic platforms for IBD [17, 20, 26–28]. Recently, nanozymes, such as Prussian blue (PB), metal–organic frameworks (MOFs), iron-based nanomaterials, and noble metal-based nanomaterials, have been widely explored for IBD treatment [28–32]. Most of these nanozymes primarily exert therapeutic effects by scavenging excessive ROS and remodeling the inflammatory microenvironment. For instance, Xianyu et al. developed a AuCe-based nanozyme enzymatic ROS scavenging abilities to against ROS-mediated damage and promote anti-inflammatory macrophage reprogramming [26]. Meanwhile, recent advances in nanozymes have reported H_2_S scavenging activity through physical adsorption, ligand coordination, or metal–sulfur bonding. For example, Cu-based nanoparticles and zirconium-based UiO-66 have been reported to exhibit H_2_S scavenging capacity and have been applied in reducing H_2_S-mediated mucin disruption and promoting intestinal barrier restoration.as H_2_S scavengers for IBD treatment [17, 20]. Despite these notable advances, few existing nanosystems can simultaneously and efficiently neutralize the two key pathogenic mediators (liquid phase ROS and gaseous H_2_S) of IBD within a single facile platform. Therefore, constructing a multifunctional nanoplatform with gas-liquid biphasic scavenging capacity is highly desirable for disrupting the self-amplified destructive VCBI loop in IBD.

**Fig. 1:**
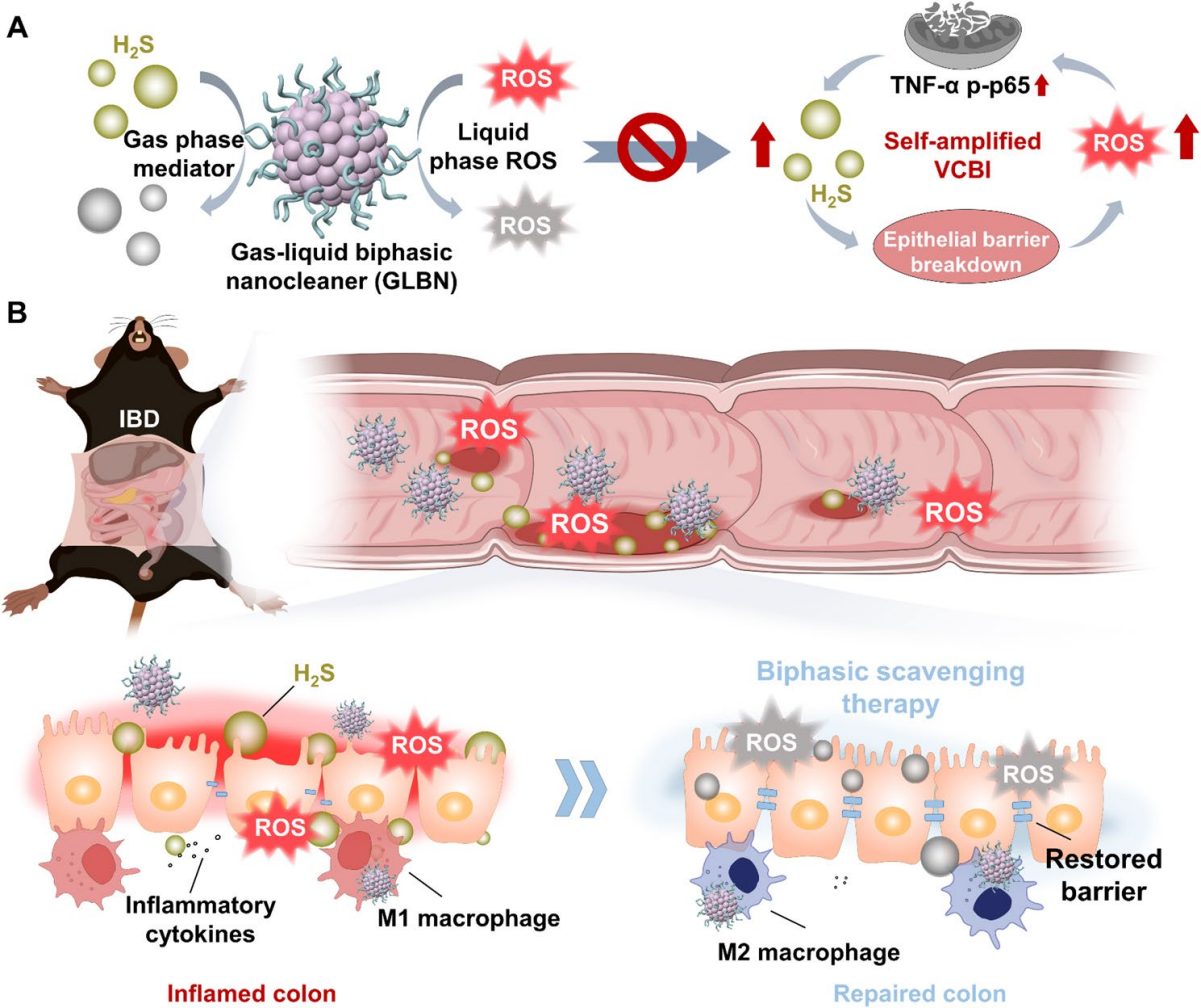
A biphasic nanocleaners GLBN is developed for highly efficient synergistic catalytic therapy of IBD by concurrent scavenging of both gas- and liquid-phase pathogenic factors. **A** GLBN disrupts the self-amplified VCBI loop through the concurrent scavenging of H_2_S and ROS. **B** The biphasic scavenging therapy of IBD by reversing gaseous H_2_S-mediated barrier damage and liquid ROS-mediated inflammatory responses

Herein, a gas-liquid biphasic nanocleaner (GLBN) is developed to disrupt the self-amplified destructive VCBI loop in IBD by concurrently scavenging the liquid ROS and gaseous H_2_S (Fig. 1). Benefiting from its high specific surface area, abundant adsorption sites, metal-sulfur bonding and ultrasmall size, the GLBN could effectively eliminate gaseous H_2_S, thereby directly mitigating H_2_S-mediated colonic barrier dysfunction. Owing to its superior intrinsic nanozyme activity, the GLBN also provides highly efficient ROS scavenging, which suppresses inflammation and promotes the beneficial repolarization of pro-inflammatory M1 macrophages toward the M2 anti-inflammatory phenotype. The origin of the biphasic scavenging capabilities, as indicated by density functional theory (DFT) calculations, is attributed to the heterogeneous facets and metal-sulfur bonding. Consequently, this desirable biphasic scavenging capacity allows the GLBN to effectively reverse the self-amplified VCBI driven by gaseous H_2_S-mediated barrier damage and liquid ROS-mediated inflammatory responses. Highly efficient therapeutic outcomes are successfully achieved and confirmed in a murine model of IBD. In summary, our work opens up a promising avenue for addressing inflammatory diseases induced by multiphase pathogenic factors (solid uric acid crystals, liquid ROS, gaseous carbon monoxide or H_2_S, etc.).

## Materials and methods

### Materials

Polyvinylpyrrolidone (PVP, Mw: 58,000Da), chloroplatinic acid hydrate (H_2_PtCl·6H_2_O), tetrazolium blue (NBT), riboflavin, methionine, Na_2_S·9H_2_O, N, N-Dimethyl-4-aminophenylamine (DMPD), Iron (III) chloride (FeCl_3_), and acetone were obtained from Aladdin Reagent (Shanghai, China). NaBH_4_ was obtained from Tianjin Kemiou Chemical Reagent Co., Ltd (Tianjin, China). Potassium titanium oxalate was bought from Macklin Biochemical Co., Ltd (Shanghai, China). RPMI 1640 medium DMEM medium and 4% paraformaldehyde were obtained from Bio-Channel Biotechnology Co., Ltd (Nanjing, China). Phosphate buffer saline solution was bought from Beijing Solab Technology Co., Ltd (Beijing, China). Hydrogen peroxide (H_2_O_2_, 30 wt%) was bought from Sinopharm Chemical Reagent Co., Ltd (Beijing, China). Sodium Dextran Sulfate (DSS) was obtained from Wuhan Saikodak Bio-Technology Co., Ltd (Wuhan, China). Lipopolysaccharides (LPS) was purchased from Sigma-Aldrich (MO, USA). Cell Titer 96 AQueous One Solution Cell Proliferation Assay Kit was bought from Promega Biotech Co., Ltd (Beijing, China). DAPI and ROS assay kit 2′−7′-dichlorofluorescin diacetate (DCFH-DA) were purchased from Beyotime Institute of Biotechnology (Shanghai, China). Mitochondrial Superoxide Detection (MitoSox) was obtained from DOJINDO Laboratories (Tokyo, Japan). 5, 5′, 6, 6′-tetrachloro-1, 1′, 3, 3′-tetraethylimidacarbocyanine iodide (JC-1) Mitochondrial Membrane Potential Assay Kit was bought from Yeasen Biotechnology Co., Ltd (Shanghai, China). Phos­phorylated p65 (p-p65) antibody (AF2006) and TNF-α antibody (AF7014) were purchased from Affinity Biosciences Co., Ltd (Jiangsu, China). Actin-Tracker Red-555 (C2203S) was purchased from Beyotime Institute of Biotechnology (Shanghai, China). Fluorescein (FITC)–conjugated Donkey Anti-Rabbit IgG (H + L) (SA00003-8) was purchased from Proteintech Group, Inc (Wuhan, China). ATP-Red 1 (GC30265) was purchased from GlpBio (CA, USA). Calcein-AM/PI Double Stain Kit (40747ES76) and Lyso-Tracker Red was purchased from Yeasen Biotechnology Co., Ltd (Shanghai, China). Fetal bovine serum (FBS) CD4 antibody (100505) were bought from Invigentech (CA, USA). Protein Carbonyl assay kit, Myeloperoxidase (MPO) assay kit and Malondialdehyde (MDA) assay kit were purchased from Nanjing Jiancheng Bioengineering Institute (Nanjing, China). ZO-1 antibody (AF5145), Occludin-1 antibody (DF7504), Claudin-1 antibody (DF6919), HIF-1α antibody (AF1009), TNF-α antibody (AF7014), iNOS antibody (AF0199), and CD163 antibody (DF8235) were purchased from Affinity Biosciences Co., Ltd (Jiangsu, China). WSP-1 was bought from Medchemexpress (MCE, USA). 8-OHdG antibody (bs-1278R), 4-hydroxynonenal (4-HNE) antibody (bs-6313R), and Foxp3 antibody (bs-10211R) were purchased from Biosynthesis Biotechnology Co., Ltd (Beijing, China). FastPure Cell/Tissue Total RNA Isolation Kit V2 (RC112-01), HiScript III All-in-one RT SuperMix Perfect for qPCR (R333-01) and ChamQ Universal SYBR qPCR Master Mix (Q711-02) were purchased from Vazyme Biotech Co., Ltd (Nanjing, China).

### Characterization

Transmission electron microscopy (TEM) and scanning transmission electron microscopy-energy dispersive X-ray spectroscopy (STEM-EDX) images were captured by FEI Tecnai F20 (FEI, USA). Dynamic light scattering (DLS) analysis was performed with Zetasizer Nano ZS90 (Malvern Instruments, UK). X-ray photoelectron spectroscopy (XPS) spectra were recorded on K-Alpha spectrometer (ThermoFisher Scientific, USA). UV-vis absorption spectra were acquired through UV-2600 spectrophotometer (Shimadzu, Japan). Fluorescence imaging was conducted using Eclipse Ti2 confocal laser scanning microscope (CLSM, Nikon, Japan). In vivo endoscopic imaging was implemented with MiniScope 2 V multifunctional soft endoscope (SHINOVA, China).

### Synthesis of GLBN

The GLBN was prepared by a one-pot method. Typically, an aqueous solution of H_2_PtCl_6_•6H_2_O (5 mL, 0.05 wt%) and polyvinylpyrrolidone (5 mL, 2.2 wt%) were mixed thoroughly at room temperature for 20 min, followed by the addition of freshly prepared NaBH_4_ (200 µL, 100 mM). The above mixture medium was stirred constantly at room temperature for 12 h to obtain a dark brown solution, after which the mixture was centrifuged (11000 rpm, 30 min) and washed with acetone to harvest the purified GLBN.

### The CAT-like activity of GLBN

To evaluate the CAT-like activity of GLBN, the H_2_O_2_ levels were detected via an ammonium titanyl oxalate assay. Briefly, 20 µL of freshly prepared H_2_O_2_ (250 mM) was mixed with 20 µL of GLBN solution at various concentrations (0, 50, 100, 200, 400 µg mL^− 1^) and 60 µL of PBS buffer at 37 °C. After incubation for 1 h, ammonium titanyl oxalate solution (200 µL, 100 mM) was added to the above mixture. The absorbance of the obtained yellow suspension at 405 nm was measured by UV-vis spectroscopy. Then, the changes of the dissolved O_2_ concentration in solution were determined to evaluate the CAT-like O_2_ production of GLBN. 1 mL GLBN solution with various concentrations (0, 50, 100, 200, 400 µg mL^− 1^) was added to 5 mL of freshly prepared H_2_O_2_ (10 mM) at room temperature, and the O_2_ level at different time points was recorded by a portable Dissolved Oxygen Meter.

### The SOD-like activity of GLBN

The SOD-like activity assay of the harvested GLBN was performed by determining the inhibition of the NBT (tetrazolium blue) photochemical reduction. In a typical experiment, NBT (22.5 µL, 10 mM), riboflavin (6 µL, 20 µM), methionine (390 µL, 0.1 M) and 750 µL GLBN solution were adjusted to a final volume of 1.5 mL with PBS buffer. Then, the reaction mixtures with sample were exposed to 365 nm UV light for 5 min. The absorbance (560 nm) of the UV treatment mixture was determined through UV-vis spectroscopy. The SOD-like activity of the sample was calculated based on the intensity of GLBN-inhibited NBT photoreduction.

### H_2_S scavenging activity of GLBN

The modified methylene-blue colorimetric method between DMPD and FeCl_3_ was used to evaluate the H_2_S levels. Briefly, freshly prepared Na_2_S (3 mM) and different concentrations of GLBN (0, 50, 100, 200, 400 µg mL^− 1^) were mixed in deionized water at 37 ℃ for 12 h. Then, 200 µL of 20 mM DMPD in 7.2 M HCl and 200 µL of 30 mM FeCl_3_ in 1.2 M HCl were added into the above solution. As the reaction proceeded, the measurement of absorbance at 660 nm was instantly detected through UV-vis spectrophotometer. To further evaluate H_2_S scavenging activity of GLBN, the same method was applied to determine the level of H_2_S at different time points (0, 2, 4, 6, 8, 10, 12 h).

### Electron spin resonance (ESR)

The ability of GLBN to scavenge hydroxyl radicals (•OH) was assessed using electron spin resonance (ESR) spectroscopy with 5,5-dimethyl-1-pyrroline N-oxide (DMPO) as a spin-trapping agent. •OH radicals were produced by a Fenton system consisting of FeCl_2_·4H_2_O and H_2_O_2_. Firstly, 200 µL of FeCl2 solution (5 mg mL^− 1^) was combined with 160 µL of deionized water and 20 µL of DMPO. The reaction commenced upon the addition of 20 µL of 30% H_2_O_2_, followed by vortexing for 5 min before ESR measurement. For the test samples, the deionized water was replaced by 160 µL of the GLBN solution, with all other steps kept unchanged.

### Cell culture

The RAW264.7 macrophages were cultured in high-glucose Dulbecco’s Modified Eagle Medium (DMEM) supplemented with 10% (v/v) fetal bovine serum (FBS). The MODE-K cells were cultured in Roswell Park Memorial Institute (RPMI) 1640 medium containing 10% (v/v) FBS and 1% (v/v) penicillin/streptomycin. All cells were incubated at 37 °C with 5% CO_2_ and 95% humidified atmosphere and purchased through commercial means.

### In vitro macrophage uptake

RAW 264.7 cells were cultured in 6-well plates or confocal dishes and incubated at 37 ℃ for 12 h. FITC-labelled GLBN was used to treat the cells for 3 h. Following this, the cells were rinsed twice with PBS, after which a serum-free medium containing LysoTracker Red dye was added to each dish, and the cellsnwere incubated at 37 ℃ for 1 h. The cells were then fixed with 4% paraformaldehyde for 25 min and rinsed three times with PBS before DAPI staining. Finally, fluorescence images were acquired using CLSM and quantified with ImageJ software.

### Cytotoxicity experiment in vitro

The RAW264.7 or MODE-K cells were seeded into 96-well plates overnight. Subsequently, the cells were treated with GLBN at various concentrations (0, 10, 20, 30, 40, 50 µg mL^− 1^) dispersed in culture medium for 12–24 h. Then, cell viability was evaluated using the Cell Titer 96 AQueous One Solution Cell Proliferation Assay (MTS assay).

### Biocompatibility test in vitro

For the biocompatibility assay of GLBN, red blood cells (RBCs) were collected from C57BL/6 mice and resuspended in physiological saline to a 5% (v/v) concentration. The freshly prepared RBC resuspension was thoroughly mixed with an equal volume of GLBN solution (25, 50, 100, 200, 400 µg mL^− 1^) in a fresh tube. The mixture was incubated at 37 °C for 1 h. Of note, RBCs treated with ddH_2_O served as the positive group, while isotonic solution-treated was the negative group. Subsequently, RBCs were collected by centrifugation (3000 rpm, 15 min) and imaged by digital camera. The absorbance of the supernatant at 570 nm was measured to calculate the relative hemolysis:

$$\begin{aligned}&\mathrm{Hemolysis} (\%) =\\& \mathrm{(A}_\mathrm{sample} - \mathrm{A}_\mathrm{negative}) / (\mathrm{A}_\mathrm{positive} - \mathrm{A}\mathrm{negative}) \times \\&100\%\end{aligned}$$

### Intracellular oxidative stress regulation experiment

The RAW 264.7 macrophages were seeded in CLSM dishes and allowed to culture overnight in high-glucose DMEM containing 10% (v/v) FBS. Then, the cells were divided into the following four groups: (1) Control; (2) LPS; (3) LPS + GLBN (10 µg mL^− 1^); (4) LPS + GLBN (50 µg mL^− 1^). All groups except the control were incubated with LPS (500 ng mL^− 1^) for 6 h for intracellular ROS generation. Subsequently, the culture medium was removed and cells were incubated with serum-free medium containing the DCFH-DA (10 µM) at 37 °C for 30 min. The cells were then fixed with 4% paraformaldehyde for 25 min and rinsed three times with PBS before DAPI staining. The intracellular ROS level was evaluated by fluorescence microscope and quantified with ImageJ software.

### Detection of intracellular H_2_S

Intracellular H_2_S level was assayed by WSP-1 staining. Briefly, The RAW 264.7 macrophages were seeded in CLSM dishes and allowed to culture overnight in high- glucose DMEM containing 10% (v/v) FBS. Then, the cells were divided into the following six groups: (1) Control; (2) LPS; (3) LPS + GLBN (10 µg mL^− 1^); (4) LPS + GLBN (50 µg mL^− 1^). All groups except the control were incubated with LPS (500 ng mL^− 1^) for 6 h for intracellular H_2_S generation. Following this, the cells were rinsed twice with PBS, after which a serum-free medium containing WSP-1 working solution was added to each dish and incubated at 37 ℃ for 30 min. The cells were then fixed with 4% paraformaldehyde for 25 min and rinsed three times with PBS before DAPI staining. Finally, fluorescence images were acquired using CLSM and quantified with ImageJ software. In order to investigate the effect of drug treatment time on H_2_S scavenging ability, the following four groups were set up: (1) Control; (2) LPS; (3) LPS + GLBN (1 h); (4) LPS + GLBN (6 h), with other steps remaining unchanged.

### Mitochondrial superoxide generation evaluation

The RAW 264.7 macrophages were seeded in CLSM dishes and cultured overnight in high-glucose DMEM containing 10% (v/v) FBS. Then, the cells were divided into the following four groups: (1) Control; (2) LPS; (3) LPS + GLBN (10 µg mL^− 1^); (4) LPS + GLBN (50 µg mL^− 1^). All groups except the control were incubated with LPS (500 ng mL^− 1^) for 6 h for intracellular mitochondrial superoxide generation. Subsequently, the cells were washed with PBS and incubated with MitoSox Red working solution (10 µM) in the dark at 37 °C for 30 min. The cell samples were then fixed with 4% paraformaldehyde for 20 min and the nuclei were stained with DAPI for 10 min. Fluorescence images were viewed using CLSM and quantified with ImageJ software.

### Mitochondrial membrane potential measurement

The RAW 264.7 macrophages were seeded in CLSM dishes and allowed to culture overnight in high-glucose DMEM containing 10% (v/v) FBS. Then, the cells were divided into the following four groups: (1) Control; (2) LPS; (3) LPS + GLBN (10 µg mL^− 1^); (4) LPS + GLBN (50 µg mL^− 1^). All groups except the control were incubated with LPS (500 ng mL^− 1^) for 6 h. Subsequently, the culture medium was replaced by freshly prepared JC-1 working solution and cells were incubated for 20 min at 37 °C. After rinsed three times with PBS, the cells were observed using CLSM and quantified with ImageJ software.

### Cell immunofluorescence staining assay

The RAW 264.7 macrophages were seeded in CLSM dishes and cultured overnight in high-glucose DMEM containing 10% (v/v) FBS. The cells were divided into the following four groups: (1) Control; (2) LPS; (3) LPS + GLBN (10 µg mL^− 1^); (4) LPS + GLBN (50 µg mL^− 1^). All groups except the control were incubated with LPS (500 ng mL^− 1^) for 6 h. Subsequently, the culture medium was removed and the cells were washed three times with PBS. Cells were then fixed with 4% paraformaldehyde for 15 min. After being washed with PBS, cells were permeabilized with 0.3% Triton X-100 for 15 min and then washed once with cold PBS. Next, 10% BSA was added to block non-specific binding sites at room temperature for 1 h and cells were incubated with the respective primary antibody (TNF-α and p-p65) at 4 ℃ overnight. After being washed thrice with PBS, cells were incubated with prepared secondary antibody at room temperature for 1 h, followed by washing three times with PBS. Then Actin-Tracker Red-555 was added to stain for 1 h and rinsed three times with PBS before DAPI staining. The immunofluorescence images were acquired using CLSM.

### Intracellular ATP level detection

MODE-K cells were seeded in 6-well plates and cultured overnight in RPMI Medium 1640 containing 10% (v/v) FBS. Cells were divided into the following four groups: (1) Control; (2) LPS; (3) LPS + GLBN (10 µg mL^− 1^); (4) LPS + GLBN (50 µg mL^− 1^). Except for the control group, all other groups were incubated with LPS (500 ng mL^− 1^) for 3 h. Subsequently, the culture medium was removed and the cells were washed gently with pre-warmed PBS. Then, cells were incubated with serum-free RPMI Medium 1640 containing ATP-Red 1 (5 µM) at 37 ℃ for 30 min in the dark. After incubation, the ATP-Red 1-containing medium was removed. The cells were washed with PBS, fixed with 4% paraformaldehyde for 25 min at room temperature and rinsed three times with PBS before DAPI staining. Finally, the fluorescence images were acquired using CLSM.

### Cell viability staining in vitro

MODE-K cells were seeded in 6-well plates and allowed to culture overnight in RPMI Medium 1640 containing 10% (v/v) FBS. Then, the cells were divided into the following four treatment groups: (1) Control; (2) LPS; (3) LPS + GLBN (10 µg mL^− 1^); (4) LPS + GLBN (50 µg mL^− 1^). All groups except the control were incubated with LPS (500 ng mL^− 1^) for 3 h. Subsequently, the culture medium was removed and the cells were washed twice with Assay Buffer (1X). The cells were immediately incubated with Calcein-AM/PI working solution (2.5 µM/4.5 µM) in serum-free medium at 37 ℃ for 30 min in the dark. Finally, the fluorescence images were acquired using CLSM.

### Real-time qPCR analysis

RAW264.7 macrophage cells were seeded in 6-well plates and cultured overnight in high-glucose DMEM containing 10% (v/v) FBS. Then, the cells were divided into the following four groups: (1) Control; (2) LPS; (3) LPS + GLBN (10 µg mL− 1); (4) LPS + GLBN (50 µg mL− 1). All other groups except the control group were incubated with LPS (500 ng mL− 1) for 6 h. After washing three times with cold PBS, all group cells were harvested and the total ribonucleic acid (RNA) was extracted using FastPure Cell/Tissue Total RNA ISOLATION kit V2. After analyzing the concentration and quality of RNA using Nanodrop, 1 µg of RNA samples were transcribed into complementary deoxyribonucleic acid (cDNA) using HiScript Ⅲ All-in-one RT SuperMix Perfect for qPCR. Quantitative real-time PCR (RT-qPCR) was performed on a CFX96 Real Time System (C1000 Touch Thermal Cycler). The relative expression levels of target genes including iNOS, TNF-α, IL-10 and Arg-1 were then normalized to Actb, and analyzed using the 2^-ΔΔCT method. Primer sequences are listed as Table S1.

### Construction of DSS-induced IBD mice model and treatments

Male C57BL/6 mice (8-week-old, 20–23 g body weight) were sourced from the Experimental Animal Center at Hangzhou Medical College, located in Hangzhou, China. Following a seven-day acclimatization period, the mice were randomly assigned to four experimental groups: (1) healthy mice receiving no treatment (Healthy), (2) IBD mice treated with PBS (IBD), (3) 5-ASA and (4) IBD mice treated with GLBN. Rectal administration of PBS, 5-ASA (10 mg kg^− 1^) or GLBN (10 mg kg^− 1^) was performed in each group of mice on days 1, 3, 5, and 7. On day 8, all mice underwent endoscopy and were subsequently euthanized. Throughout the experiment, daily monitoring of body weight changes, stool consistency, and fecal blood was conducted to calculate the disease activity index (DAI) for each mouse. On day 8, intestinal conditions were assessed using a multifunctional small animal soft endoscope to capture endoscopic images from each group. Following euthanasia, the colon length and spleen weight of each mouse were measured. Additionally, colonic tissues were harvested, sectioned, and subjected to further analyses, such as histological examination, immunofluorescence staining, MPO assay, MDA assay, and protein carbonyl assay. The Experimental Animal Ethical Committee of Hangzhou Medical College approved all animal experiments under the protocol number ZJCLA-IACUC-20,010,155.

### Histological and immunofluorescence staining of colonic tissues

Colonic tissues dissected from each group were fixed in 4% paraformaldehyde, embedded in paraffin, and sectioned into 5 μm slices for histological and immunofluorescence staining. Staining protocols included H&E, AB-PAS, and immunofluorescence targeting ZO-1, Claudin-1, Occludin-1, 8-OHdG, MPO, 4-HNE, HIF-1α, TNF-α, WSP-1, CD163, iNOS, CD4, and Foxp3. Histopathological scores were evaluated based on H&E staining, following established methods from previous studies.

### RNA sequencing and data analysis

Total RNA was isolated from colonic tissue samples of healthy mice, IBD model mice, and GLBN-treated IBD model mice. The concentration, purity, and integrity of the extracted RNA were assessed to ensure sample quality. High-quality samples (*n* = 3 per group) were selected for cDNA library preparation. Transcriptome profiling was performed on the Illumina NovaSeq 6000 platform (LC-Bio Technology, Hangzhou, China) using a paired-end 150 bp (PE150) sequencing strategy. Raw reads were filtered to remove adapters, polyA/polyG tails, and reads containing ambiguous nucleotides or low-quality bases. The resulting clean reads were aligned to the mouse reference genome GRCm39 using HISAT2. Differentially expressed genes (DEGs) were identified using the DESeq2 algorithm, with thresholds set at |log2(fold change)| > 1 and adjusted p-value < 0.05. KEGG pathway enrichment and gene set enrichment analysis (GSEA) were performed using the clusterProfiler package in R. Visualization of the results was generated using the OmicStudio platform and R software packages.

### 16S rDNA sequencing and analysis

Feces collected from each group of mice euthanized on day 8 were subjected to 16S rDNA sequencing and analysis, conducted by LC-Bio Technology Co., Ltd (Hangzhou, Zhejiang Province, China). DNA was extracted from fece samples using the E.Z.N.A.^®^ Stool DNA Kit (D4015, Omega, Inc., USA) following the manufacturer’s protocol. Subsequent steps included PCR amplification, purification of PCR products with AMPure XT beads (Beckman Coulter Genomics, Danvers, MA, USA), quantification using Qubit (Invitrogen, USA), and amplicon library preparation. Libraries were sequenced on the NovaSeq PE250 platform in accordance with the manufacturer’s guidelines. Alpha and beta diversity analyses were performed using QIIME2.

### Biosafety evaluation in vivo

Healthy C57BL/6 mice were randomly allocated into two groups: a control group receiving 200 µL PBS and a GLBN group administered 200 µL GLBN (10 mg kg^− 1^). Both treatments were delivered every other day for a total of four doses. Mice were euthanized 28 days post-treatment. To assess in vivo biosafety, major organs (heart, liver, spleen, lung, kidney, and colon) were harvested for histological evaluation using H&E staining. Additionally, serum samples were collected to measure levels of lactate dehydrogenase (LDH), creatinine (CR), blood urea nitrogen (BUN), aspartate aminotransferase (AST), alanine aminotransferase (ALT) and alkaline phosphatase (ALP), which reflect liver and kidney function.

### Statistical analysis

All statistical analyses were performed using Origin 2021. To assess significance of the difference between two group, a one-way analysis of variance (ANOVA) was tested. The 95% probability level (*p* < 0.05) represented a significant difference. LEfSe analysis (LDA score threshold: 3.5) was used for quantitative analysis of biomarkers within different groups for 16 S rDNA analysis. Data are shown as the mean ± standard deviation (S.D.). Each experiment was independently repeated at least three times (*n* ≥ 3).

## Results and discussion

### Preparation and characterization of GLBN

**Fig. 2:**
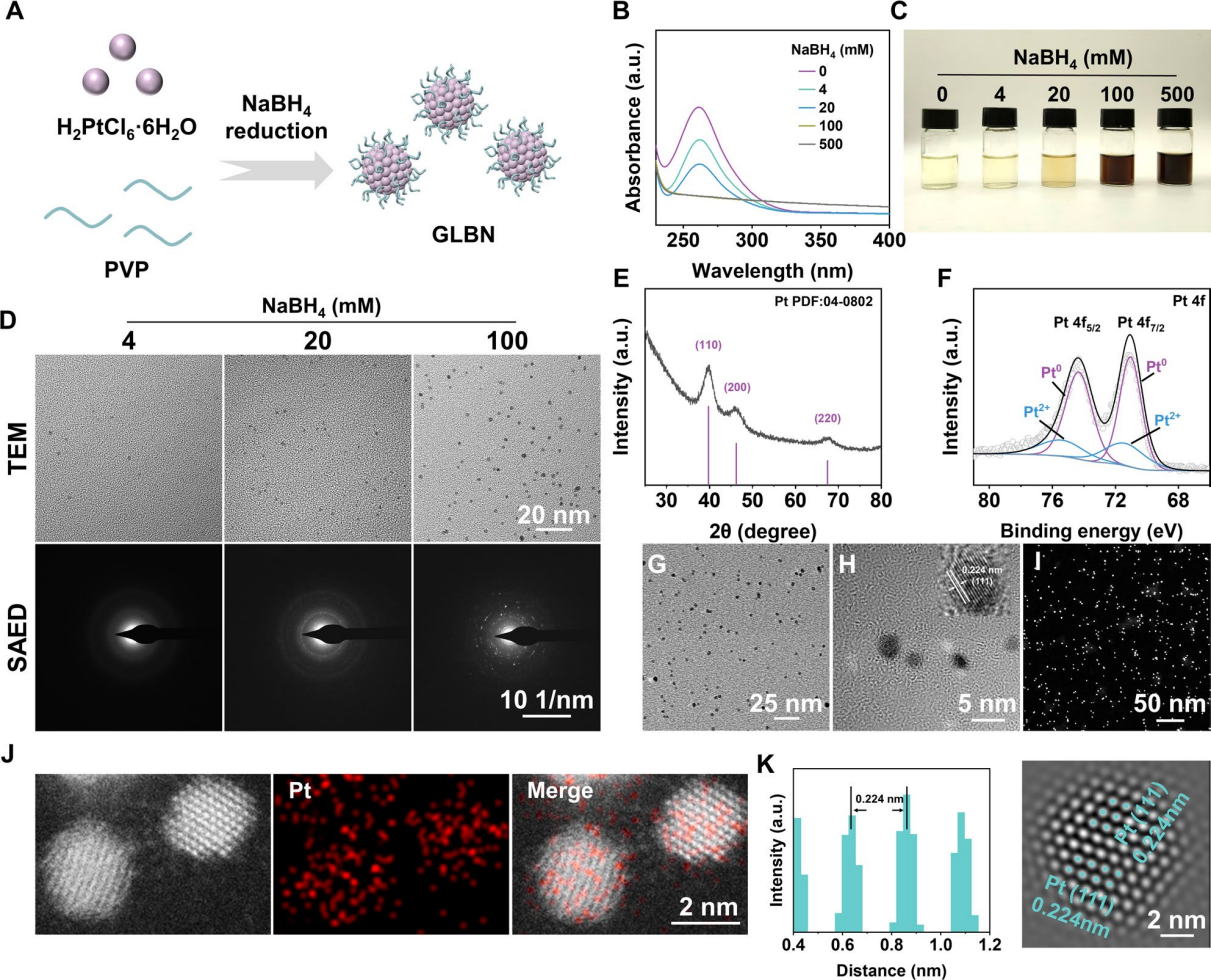
Preparation and characterization of GLBN. **A** Schematic of GLBN synthesis. **B** Ultraviolet-visible (UV-vis) spectra and **C** photographs of GLBN prepared with different NaBH_4_ concentrations. **D** TEM images and SAED patterns of GLBN with different concentrations of NaBH_4_. **E** XRD spectrum of GLBN. **F** High-resolution XPS spectra of Pt 4f in GLBN. **G** TEM image of GLBN. **H** HRTEM images of GLBN. **I** The STEM image of GLBN. **J** HAADF-STEM image and EDS elemental mapping image of GLBN. **K** Interplanar spacing pattern and iFFT micrograph of GLBN

An ultrasmall gas–liquid biphasic nanocleaner (GLBN) was synthesized via a facile one-pot aqueous NaBH_4_ reduction strategy for concurrent liquid-phase ROS scavenging and gas-phase H₂S scavenging in IBD therapy. Specifically, chloroplatinic acid hexahydrate (H_2_PtCl_6_·6H_2_O) was used as the platinum precursor and readily reduced by NaBH_4_ in the presence of polyvinylpyrrolidone (PVP), which served as a surface-capping and stabilizing agent to regulate nanoparticle formation (Fig. 2A). Given that H_2_PtCl_6_·6H_2_O solution exhibits the characteristic absorption maximum at 260 nm [33], the effect of NaBH_4_ on GLBN formation was evaluated by UV-vis spectroscopy and visual observation (Fig. 2B-C). Increasing the NaBH_4_ concentration led to obvious changes in the absorption profile and solution color, indicating a concentration-dependent formation process. Additionally, when the NaBH_4_ concentration reached 100 mM, the characteristic absorption peak of H_2_PtCl_6_·6H_2_O solution had completely disappeared, revealing that this concentration was ideal. As shown in Fig. 2D, the crystallinity and yield of GLBN are gradually enhanced with increasing NaBH_4_ concentration, which confirms that the NaBH_4_ serves as an indispensable reducing agent during GLBN formation. Meanwhile, the role of PVP is also explored. In contrast to the PVP-free sample, the product synthesized with PVP displays a monodisperse and ultrasmall nanostructure (Fig. S1). Without the PVP surface ligand, the as-obtained product suffers from obvious aggregation and precipitation (Fig. S1). Taken together, these findings validate that the strong reducing power of NaBH_4_ drives the rapid nucleation of Pt atoms, while PVP coordinates to the nascent Pt nanoparticles through its carbonyl and pyrrolidone groups, sterically inhibiting excessive particle growth and preventing aggregation, thereby yielding ultrasmall and monodisperse GLBN. In conclusion, these results revealed that NaBH_4_ played a critical role in regulating the formation process of GLBN. With the optimal synthesis conditions established, a comprehensive structural analysis of the as-obtained GLBN was performed to gain deeper insights into its structure features. As shown in Fig. 2E, the X-ray diffraction (XRD) spectrum of GLBN revealed three peaks corresponding to characteristic diffractions of Pt (110), (200) and (220), and all the XRD peaks were similar to those of Pt PDF (04–0802), confirming the stable synthesis of material. Further, GLBN was characterized by X-ray photoelectron spectroscopy (XPS) to investigate the element composition and valence state. The XPS analysis showed the appearance of strong binding energy peaks including O 1 s, N 1 s, C 1 s and Pt 4f, corresponding to PVP and Pt element in the GLBN (Fig. S2). Moreover, as demonstrated in Fig. 2F, a high-resolution spectrum of Pt 4f obtained from XPS exhibited two pairs of peaks (Pt⁰/Pt²⁺), suggesting that the surface of GLBN contained both metallic Pt and partially oxidized Pt species. To further investigate the microstructural characteristics of GLBN, transmission electron microscopy (TEM) analysis was subsequently performed. The TEM image revealed the ultrasmall size and homogeneous dispersion of GLBN (Fig. 2G) and the hydrated particle size of GLBN measured by dynamic light scattering (DLS) suggested its water-dispersibility (Fig. S3). Meanwhile, the high-resolution TEM (HRTEM) image further showed an interplanar spacing of 0.224 nm corresponding to the Pt (111) plane (Fig. 2H) [34]. Consistently, as shown in Fig. 2I, the high-angle annular dark-field scanning transmission electron microscopy (HAADF-STEM) analysis exhibited bright contrast spots corresponding to platinum atoms. Besides, the aberration-corrected STEM-EDS elemental mapping images of GLBN in Fig. 2J further confirmed the existence and uniform distribution of the Pt element. Through the inverse FFT (iFFT) reconstruction, a clear atomic arrangement closely matching the (111) crystal plane structure of Pt can be observed, intuitively confirming the presence of the Pt (111) plane in GLBN (Fig. 2K, Fig. S4). These findings collectively confirm the successful fabrication of crystalline, well-dispersed GLBN and highlight the importance of NaBH_4_ concentration and PVP stabilization in controlling its physicochemical characteristics.

### The gas-liquid biphasic scavenging capacity of GLBN

**Fig. 3:**
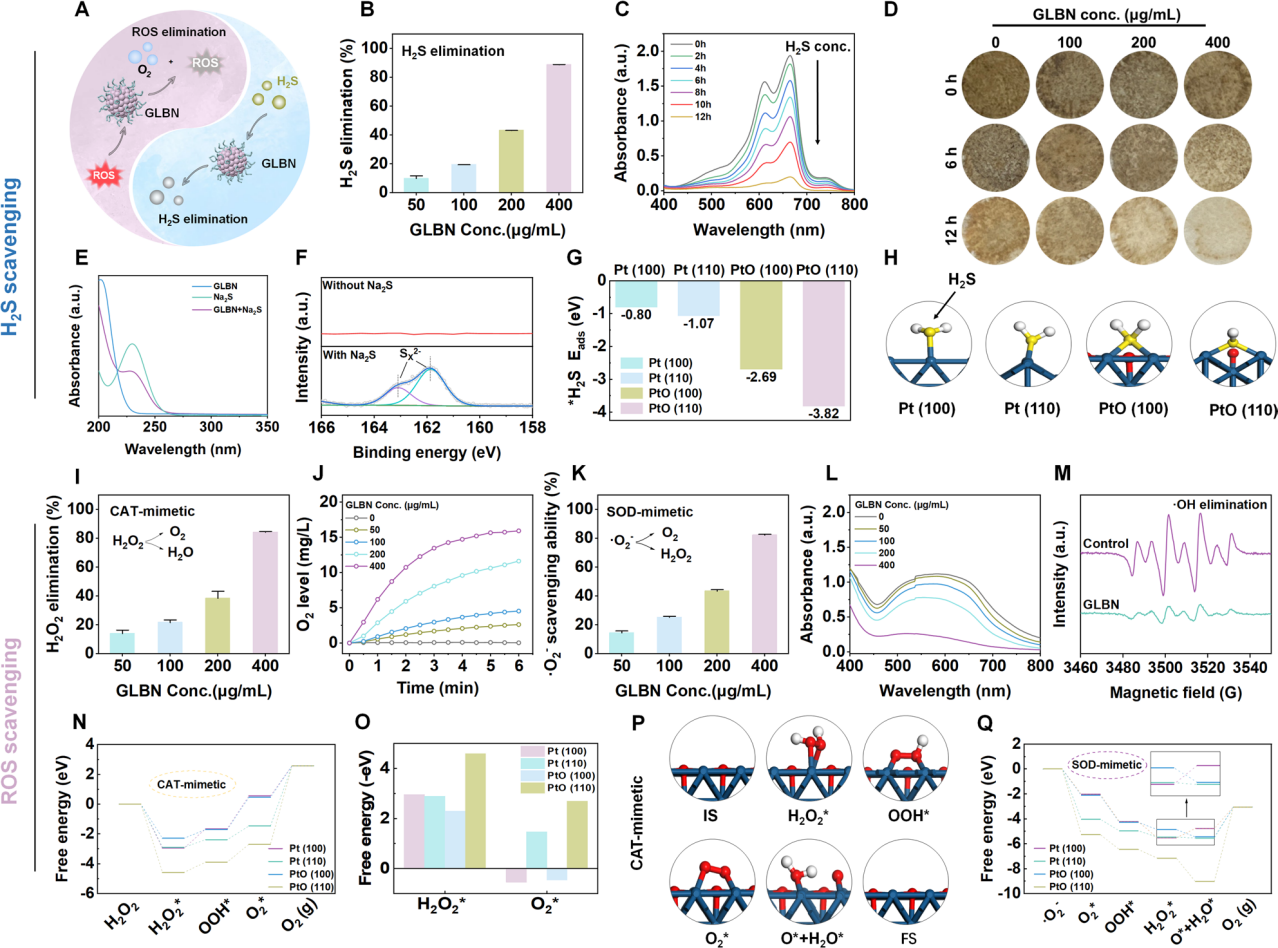
The gas-liquid biphasic scavenging capacity of GLBN. **A** Schematic of the biphasic scavenging properties of GLBN. **B** Scavenging rate of H_2_S for GLBN at different concentrations. **C** Modified MB colorimetric method for the determination of time-dependent H₂S scavenging activity of GLBN at 400 µg mL^− 1^. **D** Lead-acetate-soaked paper strips showed the elimination of H₂S after different treatments of GLBN. **E** Comparison of UV–vis spectra of GLBN with and without Na_2_S treatment. **F** S 2p XPS spectra of GLBN with and without Na_2_S treatment. **G** The free-energy diagrams of H_2_S adsorption on Pt (100), Pt (110), PtO (100) and PtO (110). **H** The heterogeneous facets and metal-sulfur bonding of GLBN. **I** CAT-mimetic activity of GLBN. **J** Generation of O_2_ by GLBN with different concentrations in H_2_O_2_ solution. **K** SOD-mimetic activity of GLBN. **L** NBT assay to determine the •O_2_^–^ scavenging activity of GLBN at different concentrations. **M** ESR spectra of the •OH-elimination by GLBN. **N** The free-energy diagrams and corresponding reaction pathways for the CAT-mimetic activities of GLBN. **O** Calculated adsorption energies of H_2_O_2_^*^ and O_2_^*^ on Pt (100), Pt (110), PtO (100), and PtO (110) surfaces. **P** Reaction pathway for the CAT-mimetic activity. **Q** The free-energy diagrams and corresponding reaction pathways for the SOD-mimetic activities of GLBN

The colonic inflammatory microenvironment features high concentrations of H₂S in the gaseous phase and ROS in the liquid phase, a hallmark that significantly exacerbates the VCBI loop. Thus, concurrently scavenging H_2_S and ROS is highly needed. As illustrated in Fig. 3A, GLBN was designed to concurrently eliminate the liquid ROS and gaseous H_2_S. We initially evaluated the H_2_S scavenging capacity of GLBN with the modified methylene-blue colorimetric method. The results revealed an excellent scavenging effect on H_2_S in a concentration-dependent manner, and the elimination efficiency increased progressively with increasing GLBN dosage, reaching nearly 90% at 400 µg mL^− 1^ (Fig. 3B, Fig. S5). As depicted in Fig. 3C and Fig. S6, this trend was further substantiated by the time-dependent spectral evolution, characterized by a progressive attenuation of the characteristic absorption peak. Additionally, the level of H_2_S after different treatments was evaluated by using lead acetate (Pb(OAc)_2_)-soaked papers, which reacted with H_2_S to form a brown lead sulfide stain. The corresponding visual colorimetric changes were presented in Fig. 3D, which indicated continuous H_2_S depletion during incubation. The exceptional capacity for hydrogen sulfide (H_2_S) capture in GLBN-related noble metal systems may be ascribed to the high affinity between noble metal nanoparticles and sulfur-bearing moieties. It is widely acknowledged that gold nanoparticles (AuNPs) exhibit a superior affinity for thiol ligands (–SH) [35, 36]. This stems from the sulfur atom donating its lone-pair electrons to form highly stable Au–S coordinate bonds with surface gold atoms [37]. Similarly, such robust metal–sulfur (M–S) interactions have also been observed in the noble metal platinum, which exhibits a strong ability to form Pt–S bonds [38, 39]. Moreover, the UV-vis spectra of GLBN changed markedly after exposure to Na_2_S compared with GLBN before exposure to Na_2_S [40], demonstrating a direct interaction between GLBN and Na_2_S (Fig. 3E). Furthermore, high-resolution XPS spectra revealed the emergence of a distinct S 2p peak after H_2_S exposure, which was absent in the pristine GLBN (Fig. 3F). DFT calculations indicated that the PtO (110) facet of GLBN possesses the minimum adsorption energy for H_2_S, identifying it as a thermodynamically preferred site for capture (Fig. 3G). The corresponding optimized adsorption configurations were shown in Fig. 3H, further supporting that oxidized Pt sites are the dominant active centers for H_2_S capture. Combined with results of DFT calculations and high-resolution XPS, it is speculated that sulfur from H_2_S could replace oxygen in the PtO (110) facet of GLBN, forming the Pt–S bonds. Furthermore, our observations align well with the findings of Shang et al. [41], who reported the treatment of the ultrasmall Pt nanoparticles with H_2_S leads to the formation of PtS nanoparticles. For ROS scavenging, we initially selected H_2_O_2_, •OH and •O_2_^−^ as representative ROS to evaluate the catalytic activity of GLBN, including superoxide dismutase (SOD)- and catalase (CAT)-mimetic activities. As depicted in Fig. 3I, GLBN demonstrated concentration-dependent CAT-mimetic activity, which efficiently neutralized more than 80% of H_2_O_2_. Meanwhile, substantial increases in oxygen were observed, indicating efficient catalytic H₂O₂ decomposition by GLBN (Fig. 3J, Fig. S7). To confirm the SOD-mimetic activity of GLBN, •O_2_^−^ scavenging experiment was performed by determining the inhibition of the NBT (nitroblue tetrazolium) photochemical reduction [42]. The results demonstrated that the percentage of •O_2_^−^ formed was significantly reduced in the presence of GLBN, showing exceptional catalytic activity in •O_2_^−^ scavenging (Fig. 3K-L). More importantly, electron spin resonance (ESR) analysis demonstrated that GLBN markedly attenuated the characteristic •OH signal compared with the control group (Fig. 3M), indicating that it could also suppress highly reactive downstream radical species. Taken together, these results demonstrate that GLBN acts as a broad-spectrum antioxidant nanozyme capable of scavenging multiple ROS simultaneously rather than regulating only a single oxidative pathway. According to previous studies, noble metal nanozymes such as platinum, ruthenium, and iridium nanozymes possess outstanding antioxidant properties, including catalase (CAT)-like and superoxide dismutase (SOD)-like activities, and have been extensively investigated in the treatment of hepatic injury [43], renal injury [44, 45], and myocardial infarction [46]. Their antioxidant capacity is primarily attributed to surface redox pairs of metal ions with variable valence states, which act as exposed active sites for the adsorption and catalytic conversion of reactive oxygen species (ROS). For instance, Ling et al. reported that oxidized Ru species in the surface of ruthenium nanozymes present a lower free energy barrier for hydrogen peroxide (H_2_O_2_) decomposition than metallic Ru, which is favorable for enhancing the catalase (CAT)-like activity [47]. To elucidate the catalytic mechanism underlying ROS scavenging capabilities of GLBN, we further analyzed the catalytic intermediates and reaction energetics on different Pt/PtO surfaces via DFT calculations. The results revealed that the CAT-like activity of the platinum nanozyme-based GLBN is dependent on surface oxidized Pt^2+^ species, and the PtO (100) facet is responsible for the efficient CAT-mimetic activity (Fig. 3N). This result was validated by the smallest overall activation barrier on PtO (100) (Fig. 3O). The optimized structural models in Fig. 3P depict the sequential adsorption states involved in the CAT-like catalytic process. Meanwhile, DFT calculations revealed that the metallic Pt (100) facet endows the GLBN with efficient SOD-mimetic performance (Fig. 3Q). Consequently, the Pt^0^/Pt^2+^ redox couple enables efficient electron transfer and active site regeneration during the cascade SOD/CAT reaction [47], which is critical for achieving continuous catalytic turnover. Taken together, the above results demonstrate that GLBN exhibits potent biphasic scavenging capacity, confirming the feasibility of using GLBN for alleviating the inflammatory responses in colitis.

### In vitro anti-inflammation property of GLBN

**Fig. 4:**
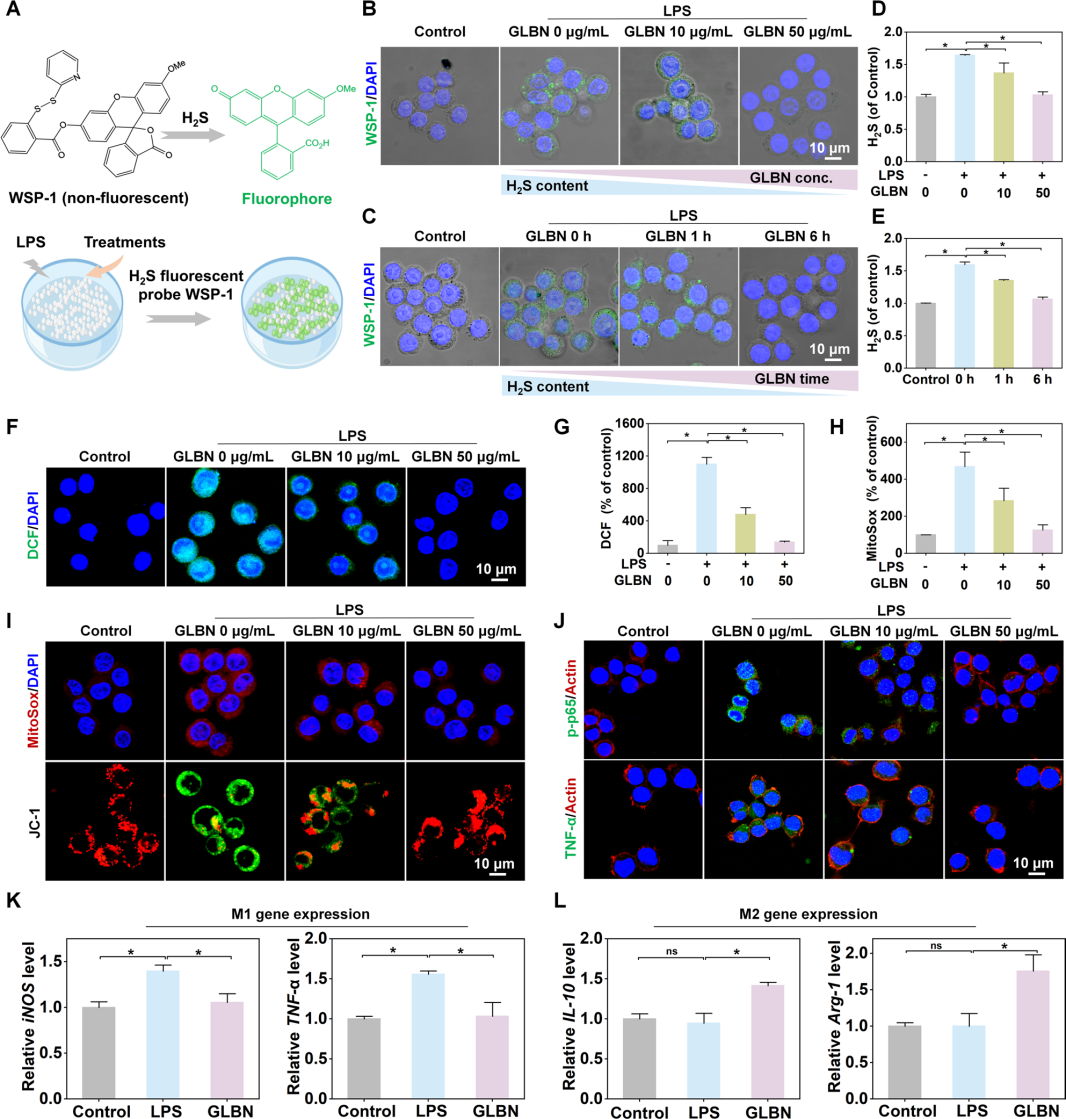
In vitro anti-inflammation property of GLBN. **A** Schematic of the WSP-1 probe-based detection of intracellular H₂S. **B** Fluorescence images of WSP-1 signal intensity in incubated cells treated with GLBN at different concentrations. **C** Fluorescence images of WSP-1 signal intensity in incubated cells treated with different times. **D** Quantitative fluorescence intensity of WSP-1 under different concentration treatments (*n* = 5). **E** Quantitative fluorescence intensity of WSP-1 under different time treatments (*n* = 5). **F** Fluorescence images of the signal intensities of DCFH-DA in cells incubated with different treatments. **G** The quantified relative fluorescence intensity of the DCFH-DA with indicated treatments (*n* = 5). **H** The quantified relative fluorescence intensity of the MitoSox with indicated treatments (*n* = 5). **I** Fluorescence images of the signal intensities of MitoSox and JC-1 in cells incubated with different treatments. **J** Fluorescent images of the immunostaining signal intensities of p-p65 and TNF-α in cells incubated with different treatments. (p-p65 and TNF-α, green channel; Nuclei, DAPI, blue channel; Actin, phalloidin-Alexa Fluor 555, red channel). **K** Relative mRNA expression levels of *iNOS* and *TNF-α* in RAW264.7 cells were measured by RT-qPCR after 6 h of LPS stimulation (*n* = 5). **L** Relative mRNA expression levels of *IL-10*, and *Arg-1* in RAW264.7 cells were measured by RT-qPCR after 6 h of LPS stimulation (*n* = 5). Data are shown as mean ± S.D. The *p* values were computed using a one-way ANOVA test. Values with *p < 0.05 are considered significant

Given that excessive gaseous H_2_S and liquid-phase ROS play critical roles in the inflammatory pathogenesis of IBD, we investigated the biphasic scavenging capacity of GLBN for these mediators in vitro. An in vitro injury model characterized by excessive ROS and H_2_S production was established using lipopolysaccharide (LPS)-stimulated RAW264.7 cells. ICP‑MS analysis of intracellular platinum content in RAW264.7 cells treated with GLBN revealed a marked increase in Pt levels, providing quantitative evidence for the efficient cellular internalization (Fig. S8). Furthermore, the intracellular delivery of GLBN was also studied using a commercial LysoTracker dye. As shown in Fig. S9, compared to the control, the green fluorescence of FITC-GLBN overlapped well with the red signal of LysoTracker dye. The readily internalized GLBN thus enabled potent intracellular scavenging of both H_2_S and ROS. To further validate that the scavenging activity targets endogenous H₂S, we performed confocal imaging using the H_2_S‑sensitive fluorescent probe WSP‑1 with an additional bright‑field channel (Fig. 4A). As presented in Fig. 4B-E, the H_2_S‑responsive WSP‑1 fluorescence was predominantly distributed inside the cell membrane, and this signal was gradually attenuated by GLBN in a dose‑ and time‑dependent manner, confirming that GLBN can effectively eliminate endogenous intracellular H_2_S. The effective clearance of intracellular H₂S significantly reversed the LPS-induced suppression of *ZO-1* gene expression; specifically, *ZO-1* mRNA abundance in MODE-K cells was markedly elevated relative to the LPS group, consequently facilitating the restoration of intestinal barrier integrity (Fig. S10). Intracellular ROS levels were detected with the ROS indicator 2′,7′-dichlorofluorescin diacetate (DCFH-DA). After GLBN treatment, the cells were stained with DCFH-DA, and the green fluorescence intensity of DCF in the LPS + 50 µg mL^− 1^ group decreased significantly compared with that in the group treated with LPS alone (Fig. 4F-G). In addition, excessive ROS production during inflammation induces oxidative damage, subsequently triggering mitochondrial dysfunction [48], while elevated H_2_S exacerbates mitochondrial injury and further amplifies ROS generation [49, 50]. We next quantitatively assessed mitochondrial ROS generation and mitochondrial membrane potential (MMP) dynamics by employing MitoSox Red probe and the MMP indicator JC-1 (5,5′,6,6′-tetrachloro-1,1′,3,3′-tetraethylbenzimidazolylcarbocyanine iodide), respectively. The results demonstrated that GLBN significantly reduced mitochondrial ROS levels and protected mitochondria from oxidative damage (Fig. 4H-I). The JC-1 probe forms a polymer or monomer in the matrix to emit red fluorescence at a higher MMP, whereas the lower MMP leads to the emission of green fluorescence [17]. The LPS-treated group alone exhibited an extensive green fluorescence, which was due to the loss of membrane potential (Fig. 4H). Notably, the MMP of the exposed cells could be fully restored at higher concentrations of GLBN (50 µg mL^− 1^), showing the protection of mitochondria. Consequently, the energy supply within the cell was significantly improved under the regulation of GLBN (Fig. S11). Moreover, MODE-K cells were utilized as an intestinal model to investigate intestinal protective effects. The cell viability was significantly restored due to the anti-apoptotic activity of GLBN (Fig. S12). Based on the above findings, we further evaluated the anti-inflammatory effects of GLBN. Phosphorylated p65 (p-p65) serves as a core activator and represents the activity marker of the NF-κB signaling pathway, while TNF-α is the down­stream inflammatory cytokine [51]. As shown in Fig. 4J, LPS stimulation significantly increased the phosphorylation of the p65 subunit (p-p65), thereby promoting the expression of TNF-α. In contrast, treatment with GLBN markedly reduced p-p65 levels and concurrently suppressed TNF-α production. Assessing the phenotypic transition of macrophages from the pro-inflammatory M1 state to the anti-inflammatory M2 state constitutes a critical determinant of anti-inflammatory efficacy. As shown in Fig. 4K-L, GLBN administration markedly downregulated the expression of M1 macrophage-specific genes, including *TNF-α* and *iNOS*, while significantly upregulating M2 macrophage-associated genes such as *Arg-1* and *IL-10*. These findings emphasize that GLBN is capable of breaking the vicious cycle driven by oxidative damage and H_2_S poisoning during inflammation, ultimately achieving gas-liquid biphasic clearance and restoration of cellular energy supply.

### Therapeutic efficacy of GLBN biphasic scavenging capacity of ROS and H_2_S against colitis

**Fig. 5:**
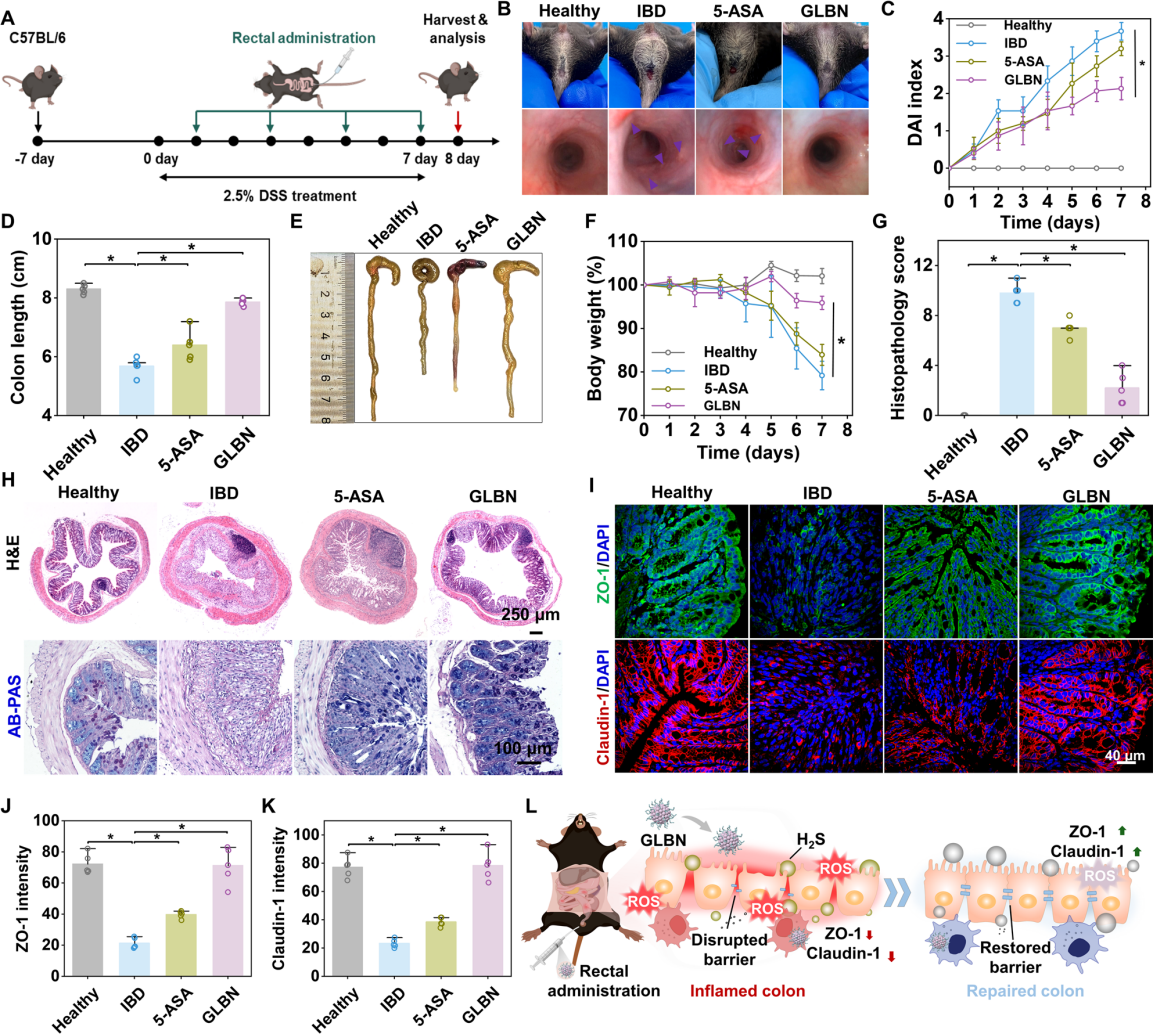
In vivo therapeutic evaluation of GLBN for intestinal barrier restoration. **A** Schematic of the experimental design. **B** Representative colonoscopy images. **C** Temporal variation trends in disease activity index (DAI) for each experimental group (*n* = 5). **D** Colon length of mice with indicated treatment (*n* = 5). **E** Digital photographs of colon of each group of mice (*n* = 5). **F** Daily changes in body weight were recorded throughout the experiment (*n* = 5). **G** Histopathological damage scores of colonic tissues (*n* = 5). **H** Representative images of colon tissues stained with H&E and AB-PAS. **I** Immunofluorescence staining for ZO-1 (green) and claudin-1 (red) proteins. **J** Quantitative analysis of ZO-1 (*n* = 5). **K** Claudin-1 protein expression levels (*n* = 5). **L** Schematic diagram of treatment efficacy for GLBN. Data are shown as mean ± S.D. The *p* values were computed using a one-way ANOVA test. Values with *p < 0.05 are considered significant

Based on the demonstrated anti-inflammatory property of GLBN in vitro, we constructed a colitis murine model using 2.5% DSS to assess the therapeutic efficacy of the sample in vivo (Fig. 5A). Before the formal therapeutic evaluation, we first performed a preliminary dose-screening study in DSS-induced colitis mice to determine the appropriate in vivo dosage of GLBN. Four doses of GLBN (0, 5, 10, and 15 mg kg^− 1^) were administered to colitis mice, and clinical and histopathological parameters were assessed. As shown in Fig. S13A-C, GLBN treatment alleviated DSS-induced disease manifestations in a dose-dependent manner, as evidenced by reduced rectal bleeding, improved body weight retention, and preservation of colon length. Additionally, the disease activity index (DAI) was used as a gold standard for assessing colitis progression, which includes body weight loss, stool consistency, and fecal bleeding. Results illustrated that the 10 mg kg^− 1^ and 15 mg kg^− 1^ groups exhibited a significantly lower DAI than the low-dose groups (Fig. S13D). To investigate the pathological damage in the colon tissues, we performed H&E (hematoxylin and eosin) and AB-PAS (Alcian blue-periodic acid Schiff) staining on the harvested colon tissue. Similarly, the low-dose groups showed structural damage to the intestine and loss of goblet cells, whereas the 10 mg kg^− 1^ and 15 mg kg^− 1^ groups maintained intact epithelial barrier structure and increased mucus-secreting glands and goblet cells (Fig. S13E). The therapeutic effect was enhanced in a dose‑dependent manner up to 10 mg kg^− 1^. However, when the dosage exceeded 10 mg kg^− 1^, the improvement in efficacy became insignificant and reached a plateau. Therefore, 10 mg kg^− 1^ was selected as the optimal therapeutic dosage of GLBN for the subsequent animal experiments.

To objectively assess the therapeutic efficacy of GLBN, 5‑aminosalicylic acid (5‑ASA), a first‑line clinical drug for the treatment of IBD, is introduced as a positive control. As shown in Fig. 5B, compared with the healthy group, the DSS-induced group exhibited severe colitis symptoms such as rectal bleeding, which were effectively mitigated by the GLBN treatment. Furthermore, endoscopic examinations demonstrated that GLBN treatment provided remarkable therapeutic efficacy against mucosal barrier damage induced by DSS (Fig. 5B). Of note, while 5-ASA conferred a discernible therapeutic benefit, its protective effect was inferior to that of GLBN. Consistent findings showed that the GLBN-treated group exhibited a significantly lower DAI (Fig. 5C) and markedly less weight loss (Fig. 5D) than the IBD group, further indicating the significant therapeutic efficacy of GLBN against DSS-induced colitis. After treatment, the colons were collected to evaluate colonic damage. We initially verified the length of colon (a main indicator for assessing IBD status), revealing that GLBN treatment effectively prevented the shortening of the colon compared to the DSS-induced group (Fig. 5E-F). As expected from H&E staining results, the colitis group showed severe structural damage compared with the healthy group, including obvious infiltration by immune cells along with epithelial and crypt damage. Although 5-ASA partially alleviated these pathological changes, GLBN treatment more markedly reversed tissue injury and significantly reduced the histopathology score to a much lower level. (Fig. 5G-H). Similarly, AB-PAS staining showed that GLBN significantly increased mucus-secreting glands and goblet cells to levels comparable to those in healthy colon tissue (Fig. 5H). Excessive ROS and H₂S are known to disrupt colonic barrier integrity by increasing mucus layer permeability and impairing the expression of key tight junction (TJ) proteins, including Zonula Occludens-1 (ZO-1), Claudin-1 and Occludin [17, 49, 52]. Therefore, we further investigated whether the biphasic scavenging capacity of GLBN could restore epithelial barrier function through modulation of TJ protein expression. Quantitative immunofluorescence analysis confirmed that GLBN treatment significantly restored colon levels of ZO-1, Claudin-1 and Occludin to those comparable to healthy controls, markedly higher than in the IBD and 5-ASA group, demonstrating that GLBN effectively protected epithelial barrier function in colitis (Fig. 5I-K and Fig. S14-15). Collectively, these results demonstrate that GLBN could effectively alleviate DSS-induced colitis and exhibited greater therapeutic efficacy than 5-ASA across key IBD indicators.

### Transcriptomic profiling reveals GLBN-mediated suppression of intestinal inflammation

**Fig. 6:**
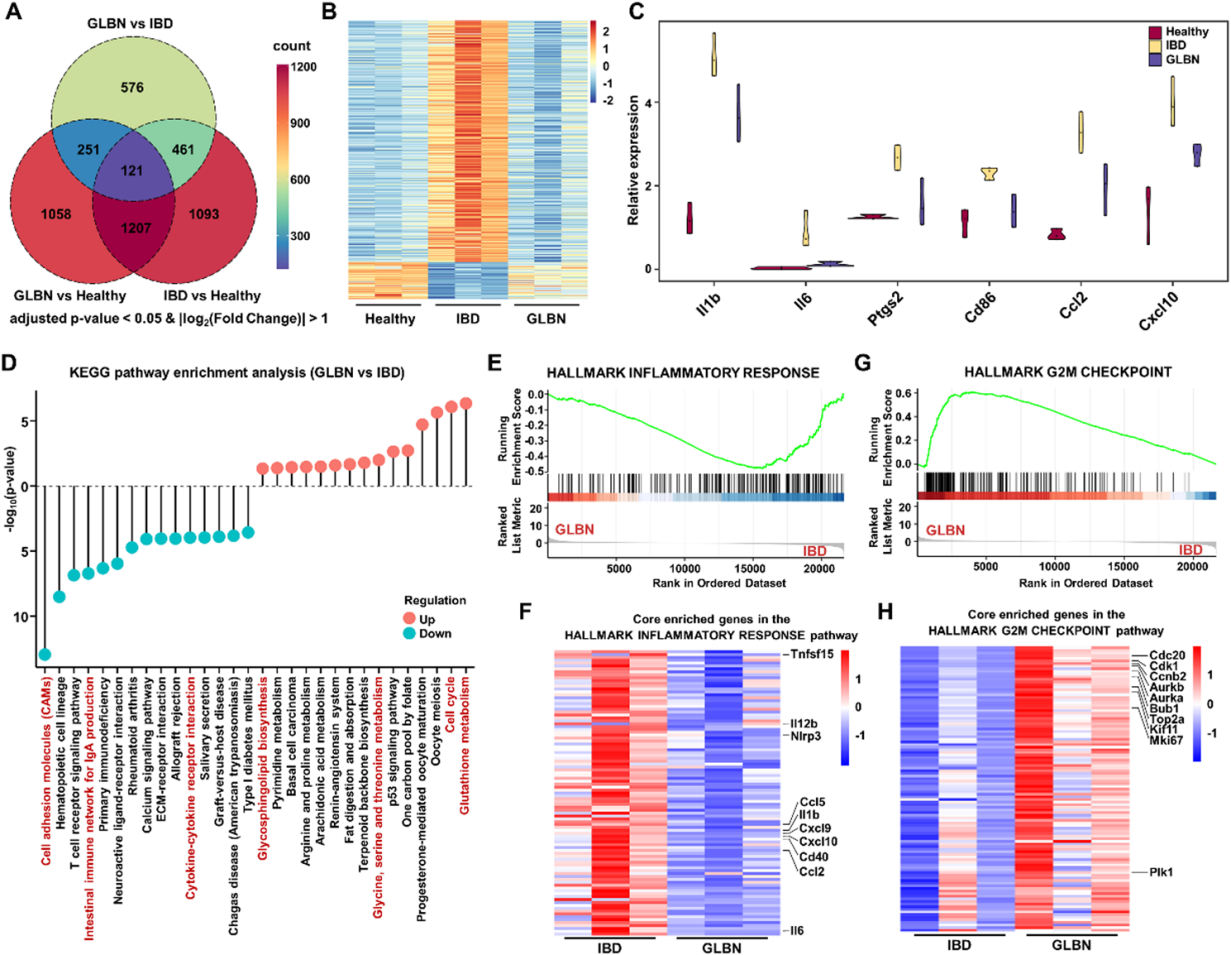
Transcriptomic analysis of colonic tissues. **A** Venn diagram showing the overlap of differentially expressed genes (DEGs) among the Healthy, IBD, and GLBN groups. DEGs were defined by adjusted p-value < 0.05 and |log_2_(Fold Change)| > 1. **B** Heatmap of upregulated and downregulated genes in different groups. **C** Relative expression levels of key inflammatory genes in the three groups. **D** KEGG pathway enrichment analysis of DEGs between GLBN and IBD groups. **E-F** GSEA of the HALLMARK inflammatory response pathway (**E**), with a heatmap of its core enriched genes (**F**). **G-H** GSEA of the HALLMARK G2M checkpoint pathway (**G**), with a heatmap of its core enriched genes (**H**)

After establishing the superior therapeutic efficacy of GLBN over the positive control, we subsequently explored its underlying therapeutic mechanisms. RNA-seq was performed on colonic tissues from healthy, IBD, and GLBN-treated IBD mice. Compared with the healthy group, 2882 differentially expressed genes (DEGs) were identified in the IBD group, while 1409 DEGs were observed between the GLBN-treated and IBD groups (Fig. 6A; Fig. S16A-B). Heatmap analysis of DEGs revealed significant intergroup differences (Fig. 6B), indicating that IBD model induction and GLBN treatment markedly altered colonic gene expression. Pro-inflammatory genes upregulated in the IBD group, including Il1b, Il6, Ptgs2, Cd86, Ccl2, and Cxcl10, were downregulated by GLBN (Fig. 6B-C), suggesting that GLBN effectively suppressed DSS-induced inflammation. Kyoto Encyclopedia of Genes and Genomes (KEGG) analysis (Fig. 6D) revealed that GLBN exerted anti‑inflammatory effects by significantly downregulating pathways, such as Cell adhesion molecules (CAMs), Cytokine‑cytokine receptor interaction, and Intestinal immune network for IgA production. In parallel, several pathways involved in cell cycle regulation were upregulated after GLBN treatment. Furthermore, gene set enrichment analysis (GSEA) results (Fig. 6E–H, Fig. S16C–D) showed that the inflammatory response pathway was strongly activated in the IBD group but effectively suppressed by GLBN treatment. In line with the KEGG results, GSEA also identified elevated signatures associated with G2M checkpoint and mitotic spindle following GLBN intervention. Collectively, these findings indicated that GLBN primarily restrains overactivated intestinal inflammatory responses. Meanwhile, the concurrent changes in cell cycle‑related pathways may contribute to the maintenance of intestinal epithelial steady state, facilitating mucosal repair. These effects together support the protective role of GLBN against IBD.

### Anti-inflammatory activity of GLBN against DSS-induced colitis

**Fig. 7:**
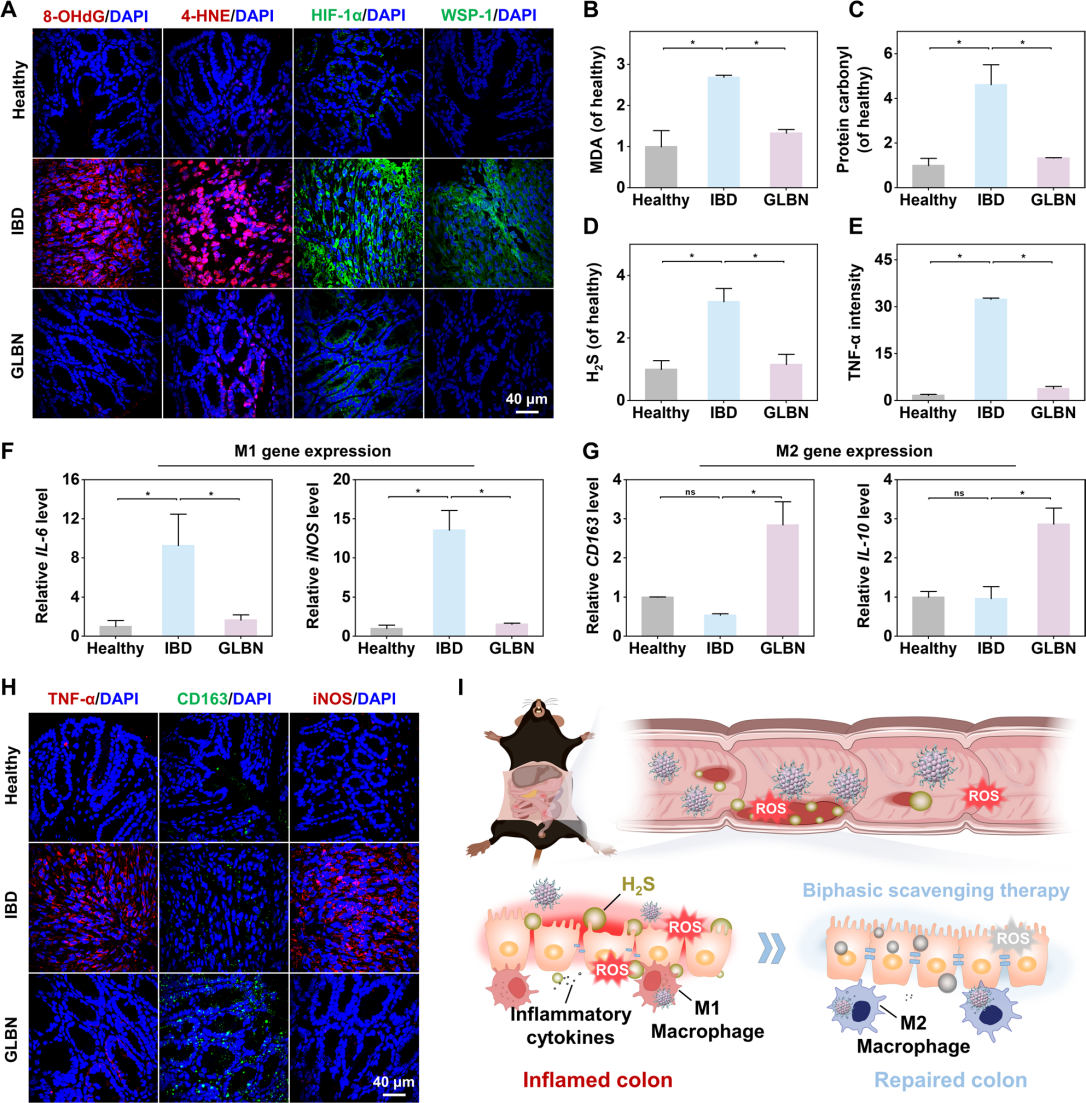
Anti-inflammatory activity of GLBN against DSS-induced colitis. **A** Immunofluorescence staining for 8-OHdG, 4-HNE, HIF-1α and WSP-l was carried out on the colon tissues after different treatments. Comparative levels of **B** MDA and **C** carbonylated protein in the colon tissues from different groups (*n* = 5). **D** Comparative levels of H_2_S in the feces excreted by mice on day 5 after various treatments (*n* = 5). **E** Quantitative immunofluorescence intensity of TNF-α (*n* = 5). **F-G** Relative mRNA expression levels of (**F**) *IL-6* and *iNOS*, (**G**) *CD163* and *IL-10* in the colon tissues were measured by RT-qPCR. (*n* = 5). **H** Immunofluorescence staining for TNF-α, CD163 and iNOS was carried out on the colon tissues after different treatments. **I** A schematic of gas-liquid biphasic scavenging therapy in IBD. Data are shown as mean ± S.D. The *p* values were computed using a one-way ANOVA test. Values with *p < 0.05 are considered significant

Encouraged by the above obtained results, the effect of GLBN against acute colitis was further evaluated by the application in the IBD mice. Given the significant role of excessive ROS and H_2_S in pathological inflammation and the biphasic scavenging capabilities of GLBN, we measured by immunofluorescence analysis the changes in redox-related markers and in the H_2_S level in colon tissue after different treatments. 8-hydroxy-2′-deoxyguanosine (8-OHdG) and 4-hydroxynonenal (4-HNE), the biomarkers of colon tissue for redox balance detection, were markedly increased in the IBD group. This indicated that the colon tissue of DSS-induced acute colitis presents severe oxidative damages, while the GLBN-treated group showed better protection (Fig. 7A). Furthermore, the expression of hypoxia-inducible factor-1α (HIF-1α), which plays a major role in the response to hypoxia, was analyzed to evaluate inflammation and tissue damage in colon tissues among different treatment groups. As illustrated in Fig. 7A, compared with the healthy group, the IBD group presented a significant hypoxic state due to inflammation and tissue damage, whereas the GLBN group showed a marked decrease in HIF-1α expression, suggesting that GLBN treatment may alleviate hypoxia. Additionally, mice treated with GLBN exhibited significantly lower malondialdehyde (MDA) and carbonylated protein levels, markers of oxidative stress in the colonic tissues, suggesting that GLBN efficiently scavenged ROS and successfully alleviated oxidative stress in the acute colitis model (Fig. 7B-C). To explore the H_2_S scavenging capacity in vivo, the WSP-1 probe was applied to detect H_2_S levels in colon tissues across different treatment groups. As demonstrated in Fig. 7A, the H_2_S levels in the colonic tissue of DSS-induced colitis mice were significantly lower than those in the IBD group and similar to the healthy group. The H_2_S scavenging capacity was also evaluated by the measurement of H_2_S levels in the feces on day 5. In IBD mice, H_2_S level increased more than 3 times in the feces, and GLBN greatly decreased the H_2_S level to normal level (Fig. 7D). In conclusion, these results demonstrate that GLBN can effectively reshape the adverse oxidative environment and reduce excessive H_2_S levels through its biphasic scavenging capacity of ROS and H_2_S in treating colitis.

Generally, tumor necrosis factor (TNF-α), a downstream pro-inflammatory cytokine in the NF-κB signaling pathway, plays a pivotal role in the pathogenesis of IBD. To evaluate the effects of GLBN treatment on inflammation, TNF-α levels in colon tissues were assessed using immunofluorescence staining. As presented in Fig. 7E, H, the expression of TNF-α in the DSS-induced colitis group was significantly higher than healthy group, indicating that DSS exacerbated inflammation in the colonic tissue. In contrast, TNF-α levels in the GLBN-treated group were significantly down-regulated compared to those in the DSS group, demonstrating the potent anti-inflammatory efficacy of GLBN treatment. This anti-inflammatory effect was further corroborated by RT-qPCR analyses showing that GLBN significantly decreased the levels of *TNF-*α and *IL-1β* compared with the untreated IBD group (Fig. S17). The expression profiles of macrophage polarization-related genes further showed that GLBN significantly downregulated the M1-associated marker *IL-6* and *iNOS* while upregulating the M2-associated markers *CD163* and *IL-10*, indicating a shift from an inflammatory M1-dominant state toward an M2-like reparative phenotype (Fig. 7F-G). Consistently, immunofluorescence co-staining for iNOS (an M1 macrophage marker) and CD163 (an M2 macrophage marker) clearly revealed that GLBN administration significantly decreased the infiltration of iNOS⁺ macrophages while increasing the proportion of CD163⁺ macrophages, suggesting a prominent phenotypic switch from M1 to M2 macrophages (Fig. 7H). To further assess the anti-inflammatory effects of GLBN, myeloperoxidase (MPO), a key marker of neutrophil infiltration, was analyzed by immunofluorescence staining and quantitative analysis in colonic tissues. As presented in Fig. S18-19, the MPO levels were greatly lower in the GLBN-treated group than IBD group, further demonstrating that GLBN treatment effectively inhibited inflammation. In addition, immunofluorescence co-staining of CD4 and Foxp3 was performed to evaluate regulatory T cell (Treg) infiltration in colonic tissues. The GLBN-treated group exhibited significantly increased Treg cell (CD4 + Foxp3+) accumulation compared to controls (Fig. S20), indicating that GLBN treatment may ameliorate intestinal inflammation through enhanced Treg-mediated immunomodulation [53]. Taken together, these results indicate that GLBN not only scavenges excessive ROS and H_2_S, but also effectively suppresses inflammation and promotes repolarization of macrophage from M1 toward M2 phenotype.

### Relative abundance of gut microbes by GLBN

**Fig. 8:**
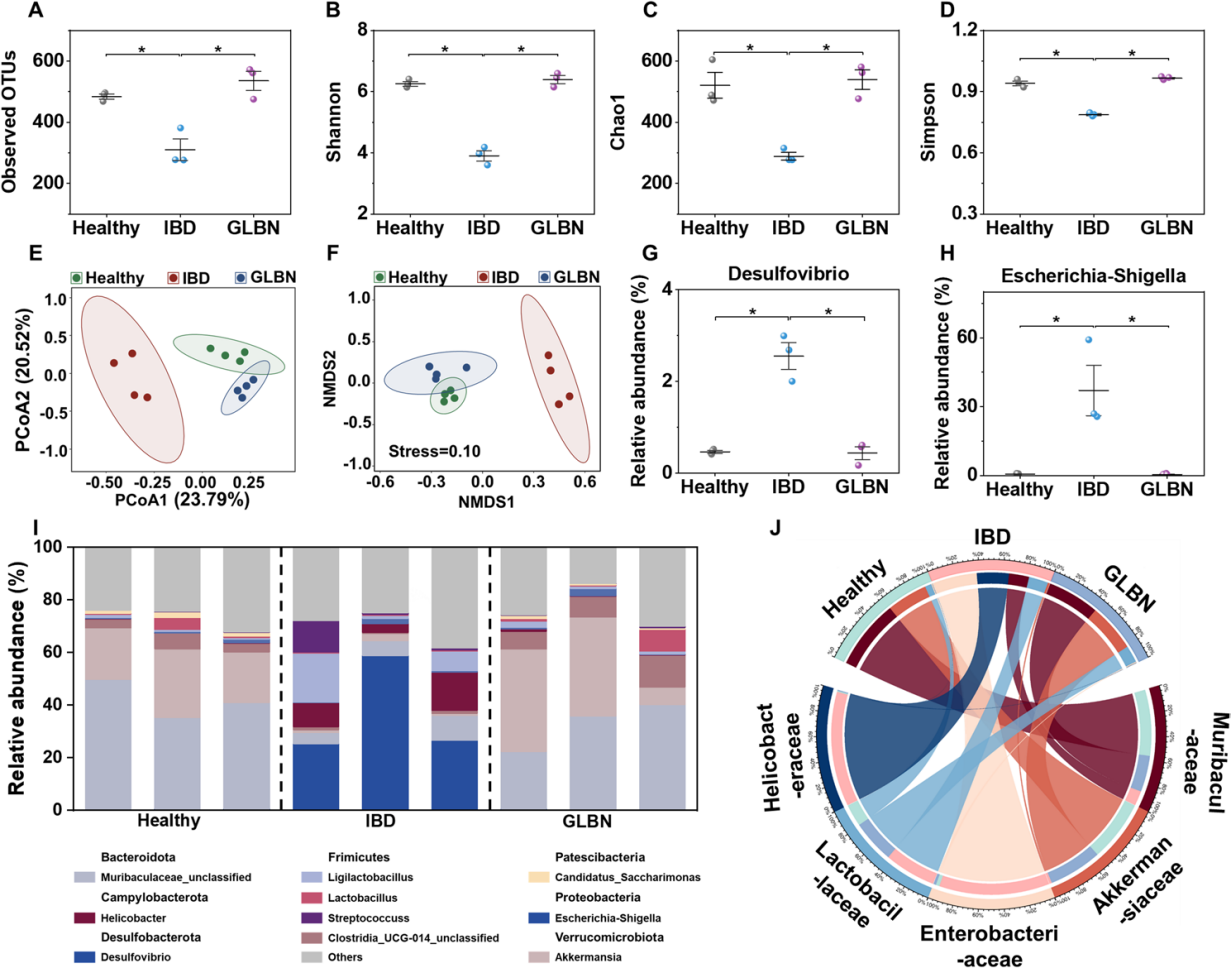
Relative abundance of gut microbes by GLBN. **A** Observed OTUs, **B** Shannon, **C** Chao1 and **D** Simpson in mice after treatment (*n* = 3). **E** PCoA and **F** NMDS analyses of the gut microbiome (*n* = 4). Relative abundance of **G***Desulfovibrio* and **H***Escherichia-Shigella* (*n* = 3). **I** Relative abundance of gut microbiome at the genus level in mice, shown as a relative percentage. **J** Chord diagram of relative abundance at the family level of gut microbiome in each mouse. Data are shown as mean ± S.D. The *p* values were computed using a one-way ANOVA test. Values with *p < 0.05 are considered significant

Disturbances in the gut microbiota are key characteristics of IBD. The gut microbiota dysbiosis plays a crucial role in excessive production of H_2_S and intestinal barrier dysfunction. To evaluate the efficacy of GLBN in modulating the gut microbiota of IBD mice, the 16 S rRNA gene sequencing analysis was conducted. Compared with the IBD group, treatment with GLBN significantly improved observed operational taxonomic units (OTUs), demonstrating a recovery of bacterial richness (Fig. 8A). In addition, the bacterial community diversity was significantly reduced by calculating the Shannon, Chao1 and Simpson indices in DSS-induced colitis mice, while GLBN treatment increased microbial diversity (Fig. 8B-D). Furthermore, the β-diversity was evaluated to investigate the changes in gut microbiota composition via the principal coordinate analysis (PCoA) and nonmetric multidimensional scaling (NMDS). The diagrams indicated that the microbial community structure in the GLBN group was similar to that of the healthy group (Fig. 8E-F). The comparison result demonstrated that the GLBN group exhibited a decrease in harmful bacteria, such as *Desulfovibrio* and *Escherichia-Shigella* (Fig. 8G-H), while simultaneously increasing beneficial bacteria, such as *Muribaculaceae-unclassified* and *Akkermansia* (Fig. S21-22). Interestingly, the level of a sulfate-reducing bacteria, *Desulfovibrio*, recovered to normal after the GLBN treatment, thus avoiding the excessive H_2_S gas production in the intestinal lumen milieu [54]. Furthermore, to investigate the composition of the microbiota, bar charts were generated to assess the key bacterial groups at the genus level in mice, shown as a relative percentage. The GLBN treatment significantly altered microbial diversity and composition and restored gut microbiome dysbiosis, increasing the relative abundance of beneficial bacteria (e.g., *Muribaculaceae_unclassified*, *Akkermansia*, and *Clostridia_UCG-014_unclassified*) and decreasing that of harmful bacteria (e.g., *Escherichia-Shigella* and *Helicobacter*) (Fig. 8I). Moreover, the relative abundance of beneficial bacteria, such as *Muribaculaceae*, *Akkermansiaceae* and *Lactobacillaceae* was recovered after GLBN treatment, demonstrating the crucial role of GLBN in restoring the healthy gut microbiota (Fig. 8J).

### Biocompatibility of GLBN therapy

**Fig. 9:**
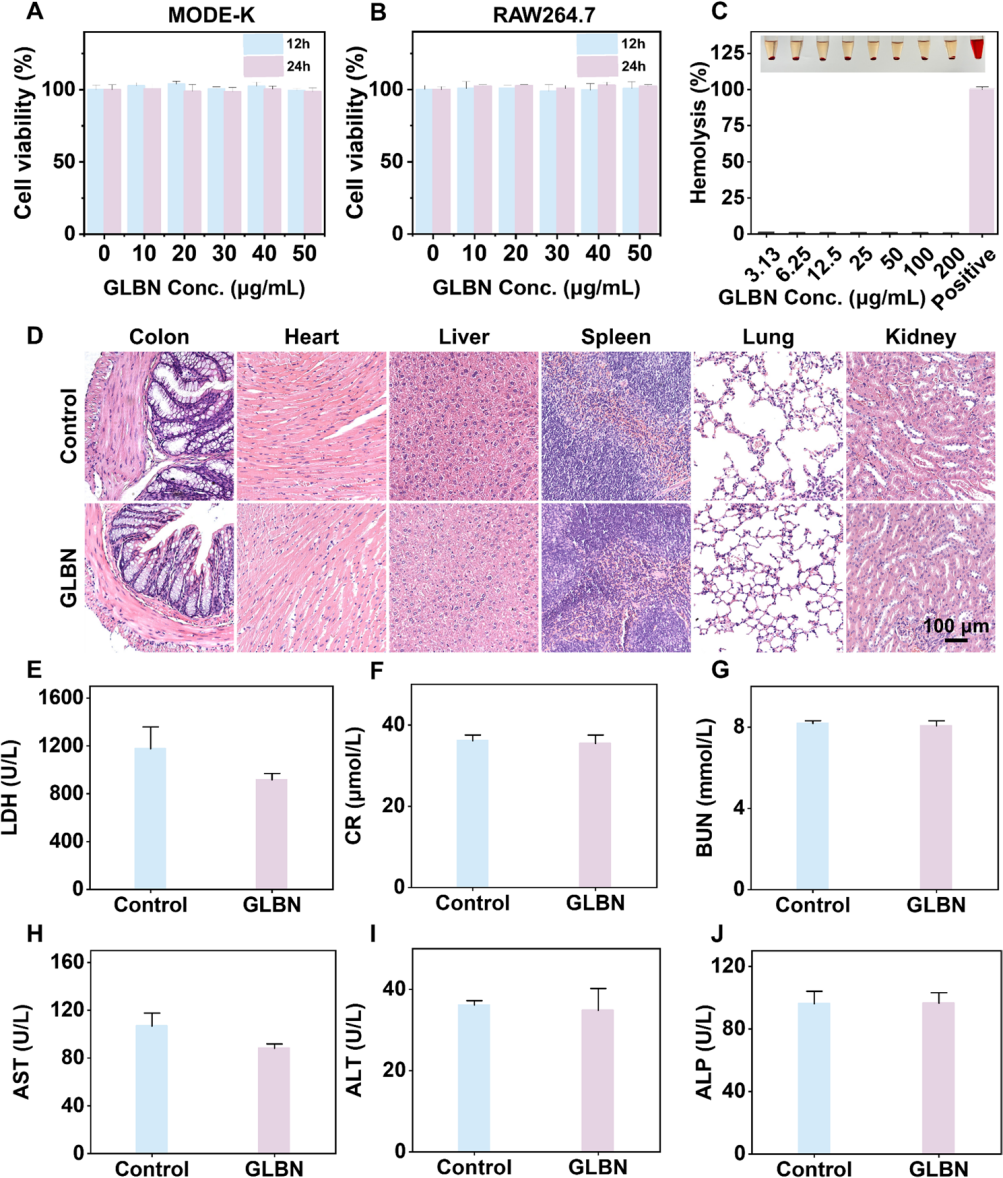
Preliminary toxicity assessment of GLBN. Cell viability at different concentrations of GLBN after co-culture with **A** MODE-K and **B** RAW264.7 cells for 12 h and 24 h. **C** Hemolysis rate at different concentrations of GLBN. **D** Histopathological images by H&E of the heart, liver, spleen, lung, and kidney from C57BL/6 mice treated for 28 days to assess systemic toxicity. Scale bar = 100 μm. **E-J** Blood biochemistry analysis of C57BL/6 mice in different groups on day 28 (*n* = 3). (**E**) LDH, Lactate dehydrogenase; (**F**) CR, creatinine; (**G**) BUN, blood urea nitrogen; (**H**) AST, aspartate aminotransferase; (**I**) ALT, alanine aminotransferase; (**J**) ALP, alkaline phosphatase. Data are shown as mean ± S.D

Encouraged by the excellent efficacy of GLBN, the biocompatibility and biosafety of GLBN were evaluated to confirm the potential for the clinical translation of GLBN. In brief, the in vitro biocompatibility of GLBN was evaluated in RAW264.7 cells and MODE-K epithelial cells. As shown in Fig. 9A and B, cells treated with increasing concentrations of GLBN (from 0 to 50 µg mL^− 1^) for 12 h and 24 h exhibited high biocompatibility in terms of cell viability. Furthermore, no significant hemolysis was observed in the hemolysis assay, indicating that GLBN exhibited favorable in vitro biocompatibility (Fig. 9C). The biosafety and biocompatibility of GLBN were further verified at the animal level. Mice were treated with GLBN (10 mg kg^− 1^) every other day for 4 times in total and were euthanized 28 days after treatment to evaluate the impact of GLBN on the structure and function of major organs and the blood system. H&E staining revealed no detectable damage or histopathological lesions in the major organs after GLBN treatment (Fig. 9D). Examination of related indices of liver and kidney function showed that GLBN did not affect lactate dehydrogenase (LDH), creatinine (CR), blood urea nitrogen (BUN), aspartate aminotransferase (AST), alanine aminotransferase (ALT) and alkaline phosphatase (ALP) (Fig. 9E-J). Taken together, the above results show that GLBN exhibited excellent biocompatibility and biosafety both in vitro and in vivo.

## Conclusion

IBD is a chronic, recurrent inflammatory disorder characterized by multifactorial pathogenesis that results in a lifelong medical burden on families. Recent studies indicate that the relapsing mechanisms of IBD are mainly driven by the self-amplified VCBI, in which multiple pathogenic mediators in different physical phases intertwine in complex ways, posing a significant challenge in long-term remission. In this study, aiming to address the challenges of the self-amplified VCBI, a gas-liquid biphasic nanocleaner GLBN is developed to concurrently eliminate the pathogenic factors derived from both liquid and gaseous phases. Remarkably, the experimental studies and theoretical evidence determined that the innovative nanocleaner exhibits unique heterogeneous facets, endowing it to present superior SOD/CAT-mimetic activities for efficient ROS levels mitigation, which eventually inhibits ROS-driven inflammatory and downregulates the proportion of M1/M2 macrophages. Moreover, GLBN-facilitated excellent gaseous H₂S eliminating by the formation of metal-sulfur bonds further mitigates H_2_S-mediated damage to the colonic barrier. Benefiting from these scavenging attributes of GLBN in both liquid phase and gas phase, in vivo and in vitro experimental results demonstrate that it exhibits sustained therapeutic efficacy for IBD, leading to remarkable mitigation of inflammatory symptoms, preservation of intestinal barrier and restoration of intestinal microbiota balance. Compared with current therapeutics, which mainly target single mediator, our innovative strategy offers a novel paradigm for addressing inflammatory diseases induced by multiphase pathogenic factors (solid uric acid crystals, liquid ROS, gaseous carbon monoxide or H_2_S, etc.) and shows significant clinical translation potential. Future research should focus on evaluating long-term safety and translating into practical treatment options.

## Supplementary information

Supplementary material 1

## Data Availability

Data will be made available on request.
