# Supplementary material for: A gas-liquid biphasic nanocleaner for breaking the self-amplified vicious cycle of barrier disruption-inflammation in inflammatory bowel disease
Source: J Nanobiotechnology. 2026 May 24;24:681. doi: 10.1186/s12951-026-04581-1 (PMC13383427; doi:10.1186/s12951-026-04581-1)
Supplement: Supplementary file 1 — Supplementary material 1 [file 12951_2026_4581_MOESM1_ESM.docx]

**Supporting Information**

**A Gas-Liquid Biphasic Nanocleaner for Breaking the Self-Amplified Vicious Cycle of Barrier Disruption-Inflammation in Inflammatory Bowel Disease**

Haibin Wu ^1,2^^†^*, Jixiang Zhang ^1†^, Xinqing Huang ^1†^, Bo Zhang ^3†^, Yuting Xie ^1^, Qingyu Li ^1^, Li Yang ^1^, Weiqi Wu ^1^, Ziying Zheng ^1^, Ziyan Huang ^1^, Fanru Gao ^1^, Zhongqing Cai ^1^, Jiahe Chen ^1^, Guohuan Zeng ^1^, Daishun Ling ^3^*, Guang Liang ^1,2^*, Qian Chen ^1,4^*

*^1^ School of Pharmaceutical Sciences, Hangzhou Medical College, Hangzhou, 311399, Zhejiang, China*

*^2^ Department of Pharmacy and Institute of Inflammation, Zhejiang Provincial People's Hospital, Affiliated People's Hospital, Hangzhou Medical College, Hangzhou, 310014, Zhejiang, China*

*^3^ Frontiers Science Center for Transformative Molecules, School of Chemistry and Chemical Engineering, School of Biomedical Engineering, National Center for Translational Medicine, Shanghai Advanced Research Institute, Shanghai Jiao Tong University, Shanghai, 200240, China*

*^4^ State Key Laboratory of Chinese Medicine Modernization, Innovation Center of Yangtze River Delta, Zhejiang University, Jiaxing, 314102, Zhejiang, China*

*Corresponding authors.

*E-mail addresses:* wuhaibin@hmc.edu.cn (H. Wu), dsling@sjtu.edu.cn (D. Ling), wzmcliangguang@163.com (G. Liang), chenqian08@zju.edu.cn (Q. Chen).

^†^ Haibin Wu, Jixiang Zhang, Xinqing Huang and Bo Zhang contributed equally.

**Supplementary Figures**


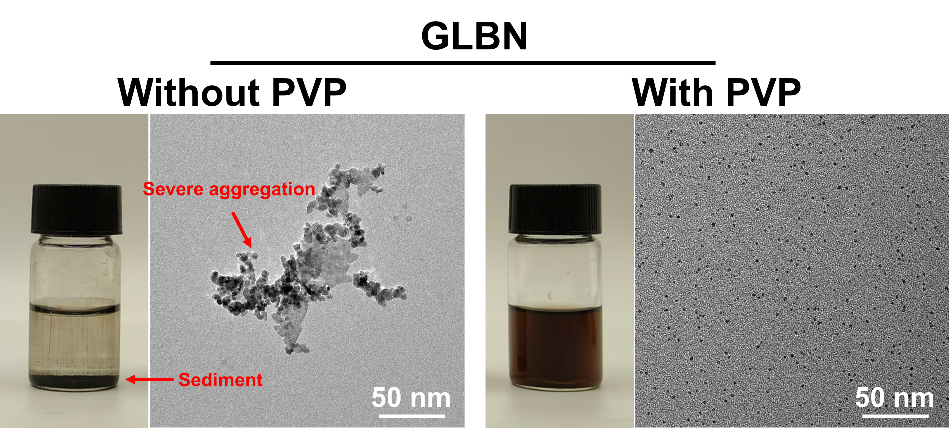


**Fig. S1** Photographs and TEM images of GLBN prepared without or with PVP.

**Fig. S2** XPS spectra of GLBN.

**Fig. S3** DLS characterization of GLBN.


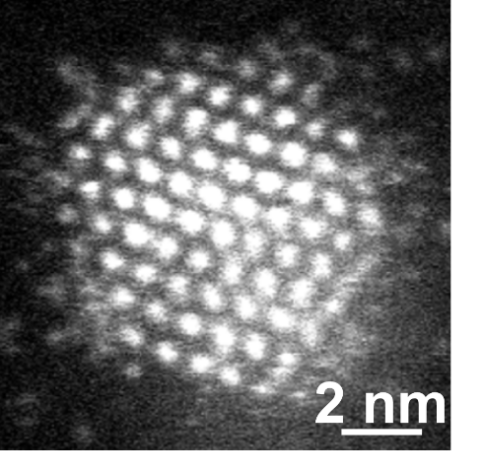


**Fig. S4** HRTEM image of GLBN.

**Fig. S5** Modified MB colorimetric method for the determination of dose-dependent H₂S scavenging activity of GLBN at 400 μg mL^-1^.

**Fig. S6** Comparison of H_2_S scavenging rates with and without GLBN treatment.


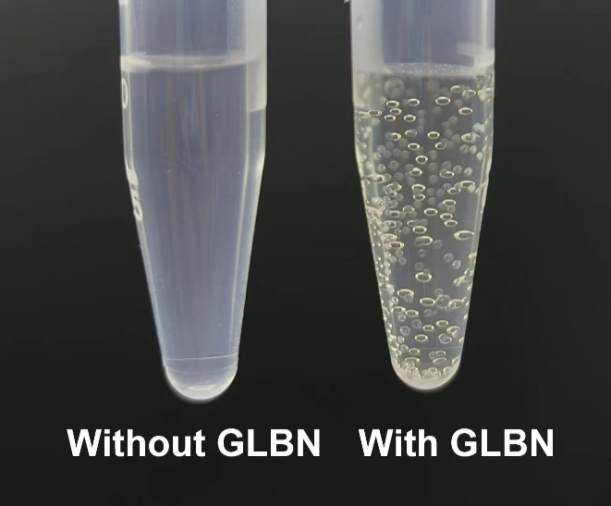


**Fig. S7** Generation of O_2_ by GLBN in H_2_O_2_ solution.

**Fig. S8** Intracellular platinum content detected by ICP-MS after incubation with blank control or GLBN (n = 3). Data are shown as mean ± S.D. *p* values were calculated via one-way ANOVA test. **p* < 0.05.

**
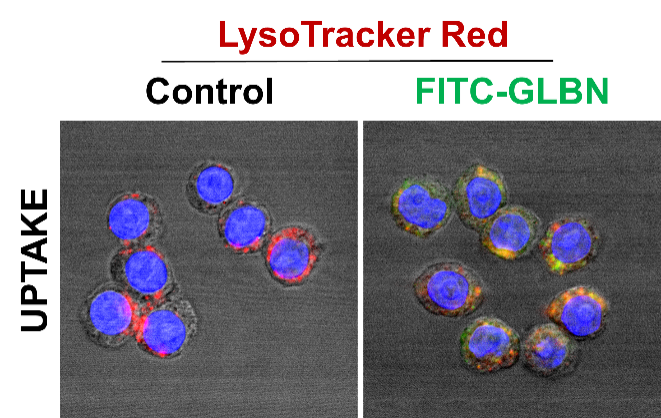
**

**Fig. S9** CLSM images of cellular uptake of FITC-tagged GLBN, the lysosomes are stained by the LysoTracker Red dye.


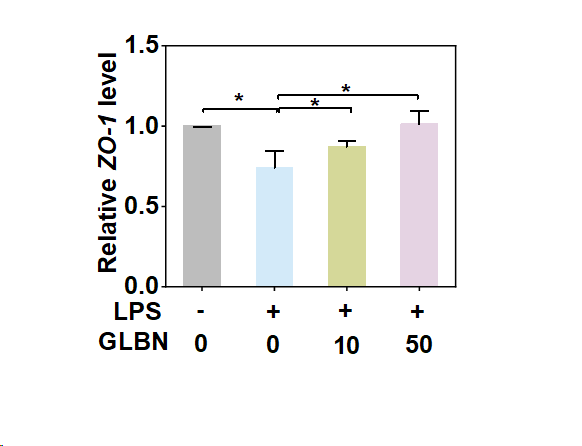


**Fig. S10** Relative mRNA expression levels of *ZO*-1 in RAW264.7 cells were measured by RT-qPCR after 6 h of LPS stimulation (n = 5). Data are shown as mean ± S.D. *p* values were calculated via one-way ANOVA test. **p* < 0.05.


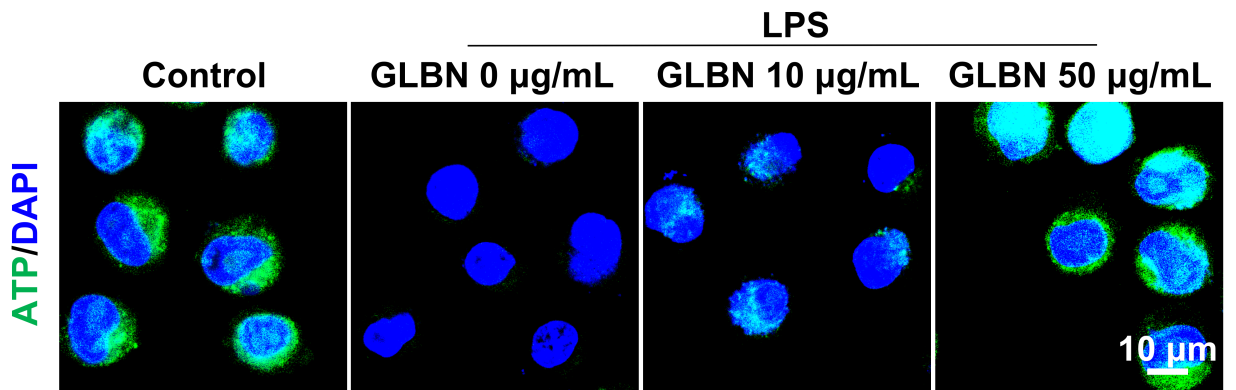


**Fig. S1****1** Fluorescence images of the signal intensities of ATP in cells incubated with different treatments.


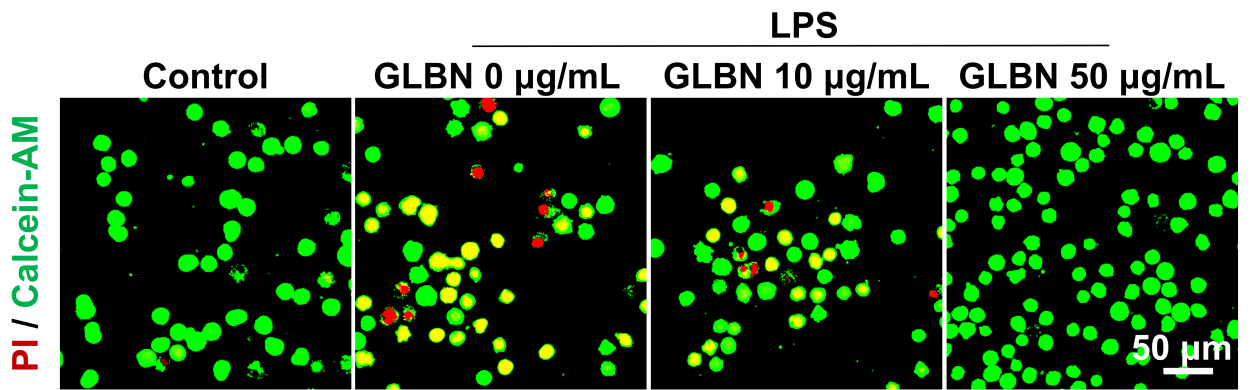


**Fig. S12** Fluorescence images of cell viability staining in cells incubated with different treatments.

**
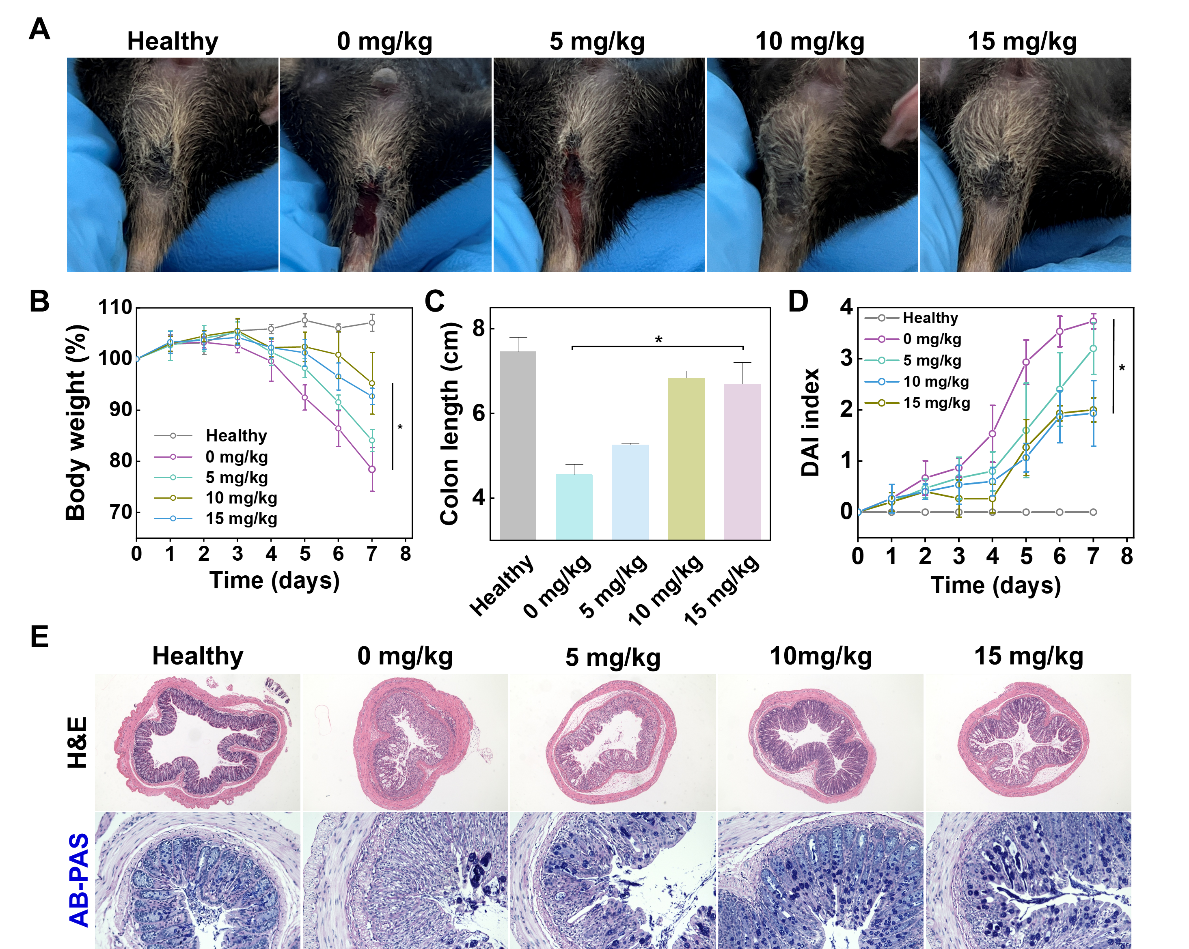
**

**Fig. S13** **A** Visual comparison of rectal bleeding in different treatment groups. **B** Daily changes in body weight recorded throughout the experiment (n = 5). **C** Colon lengths of mice with indicated treatment (n = 5). **D** Temporal trends in disease activity index (DAI) for each experimental group (n = 5). **E** Representative images of colon tissues stained with H&E and AB-PAS. Data are shown as mean ± S.D. *p* values were calculated via one-way ANOVA test. **p* < 0.05.


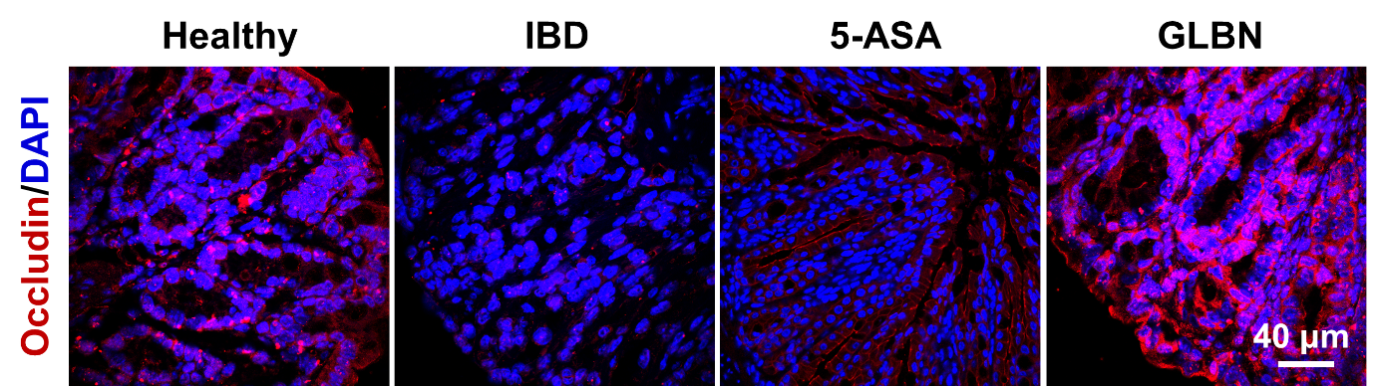


**Fig. S14** Immunofluorescence staining for Occludin was carried out on the colon tissues after different treatments.

**Fig. S15** Corresponding quantitative analysis of average fluorescence intensity of Occludin in the colon tissues of each group (n = 5). Data are shown as mean ± S.D. *p* values were calculated via one-way ANOVA test. **p* < 0.05.


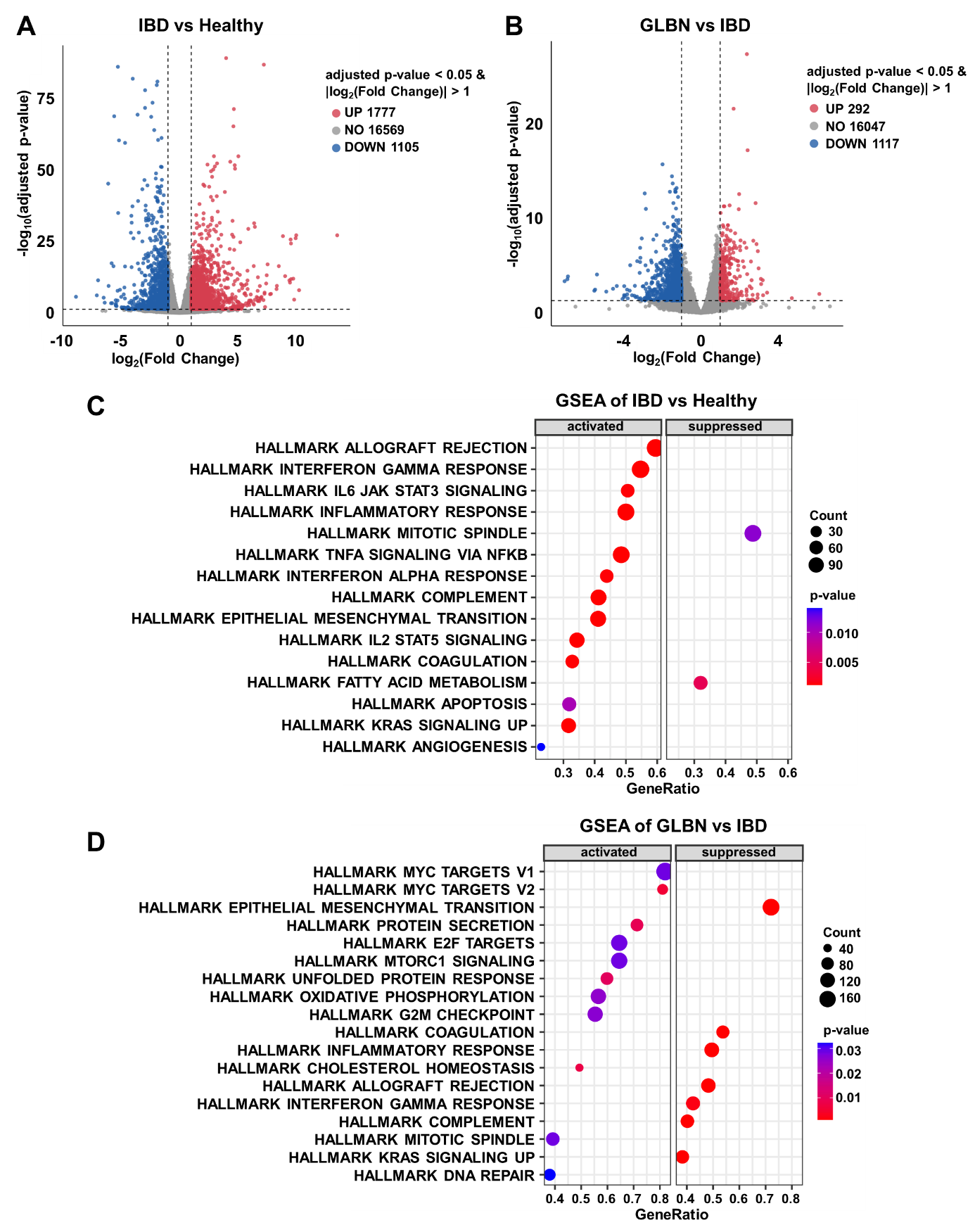


**Fig. S16 A-B** Volcano plots of differentially expressed genes (DEGs) in (**A**) IBD vs Healthy and (**B**) GLBN vs IBD. DEGs were defined by adjusted p-value < 0.05 and |log2(Fold Change)| > 1. **C-D** Dot plots of GSEA-enriched HALLMARK pathways from (**C**) IBD vs Healthy and (**D**) GLBN vs IBD, where color indicates p-value and dot size corresponds to gene count.


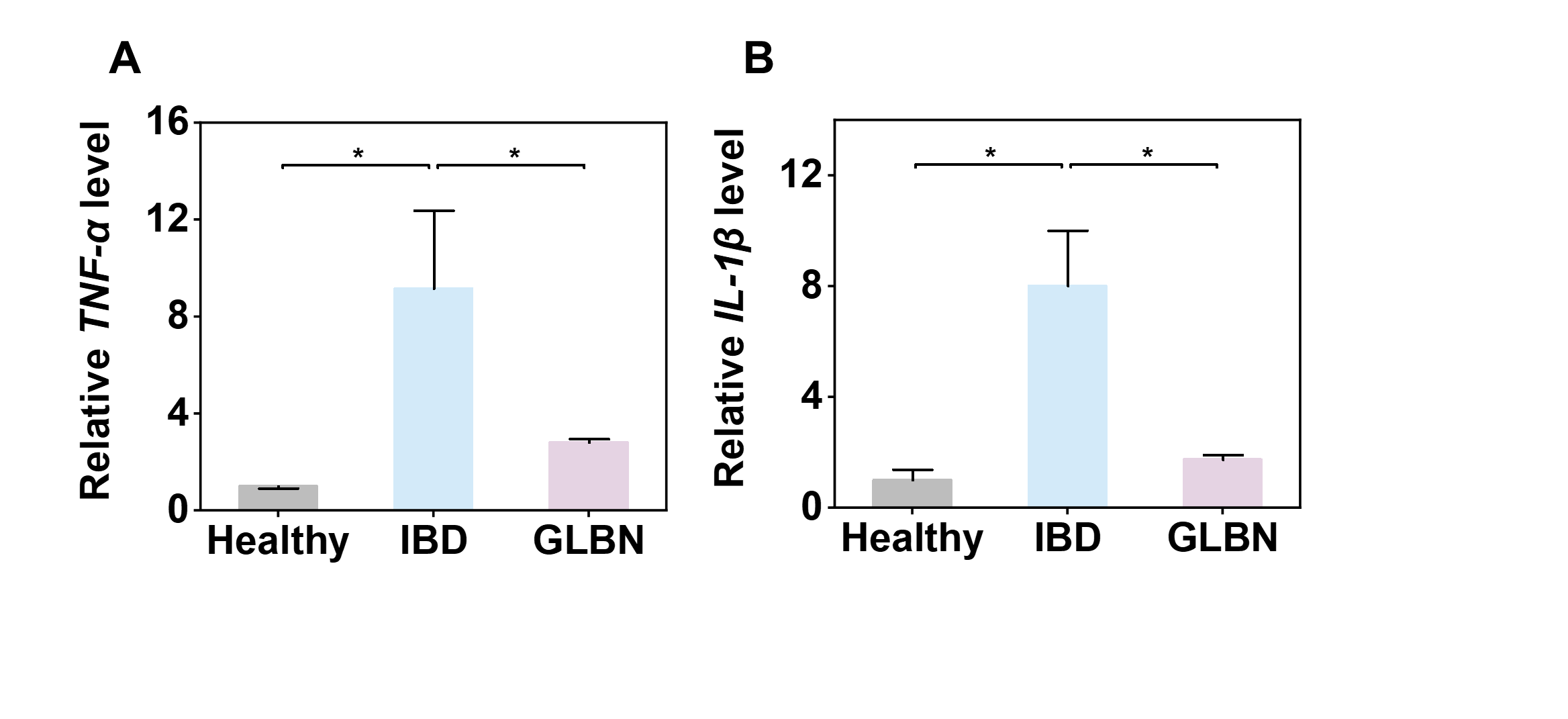


**Fig. S17** Corresponding quantitative analysis of average fluorescence intensity of (**A**) *TNF-α* and (**B**) *IL-1β* in the colon tissues of each group (n = 5). Data are shown as mean ± S.D. *p* values were calculated via one-way ANOVA test. **p* < 0.05.


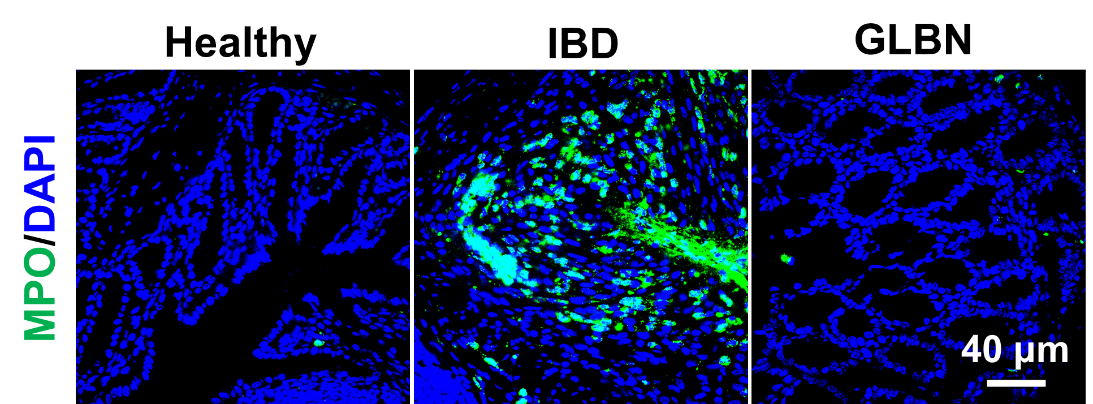


**Fig. S18** Immunofluorescence staining for MPO was carried out on the colon tissues after different treatments.

**Fig. S19** Corresponding quantitative analysis of average fluorescence intensity of MPO in the colon tissues of each group (n = 5). Data are shown as mean ± S.D. *p* values were calculated via one-way ANOVA test. **p* < 0.05.


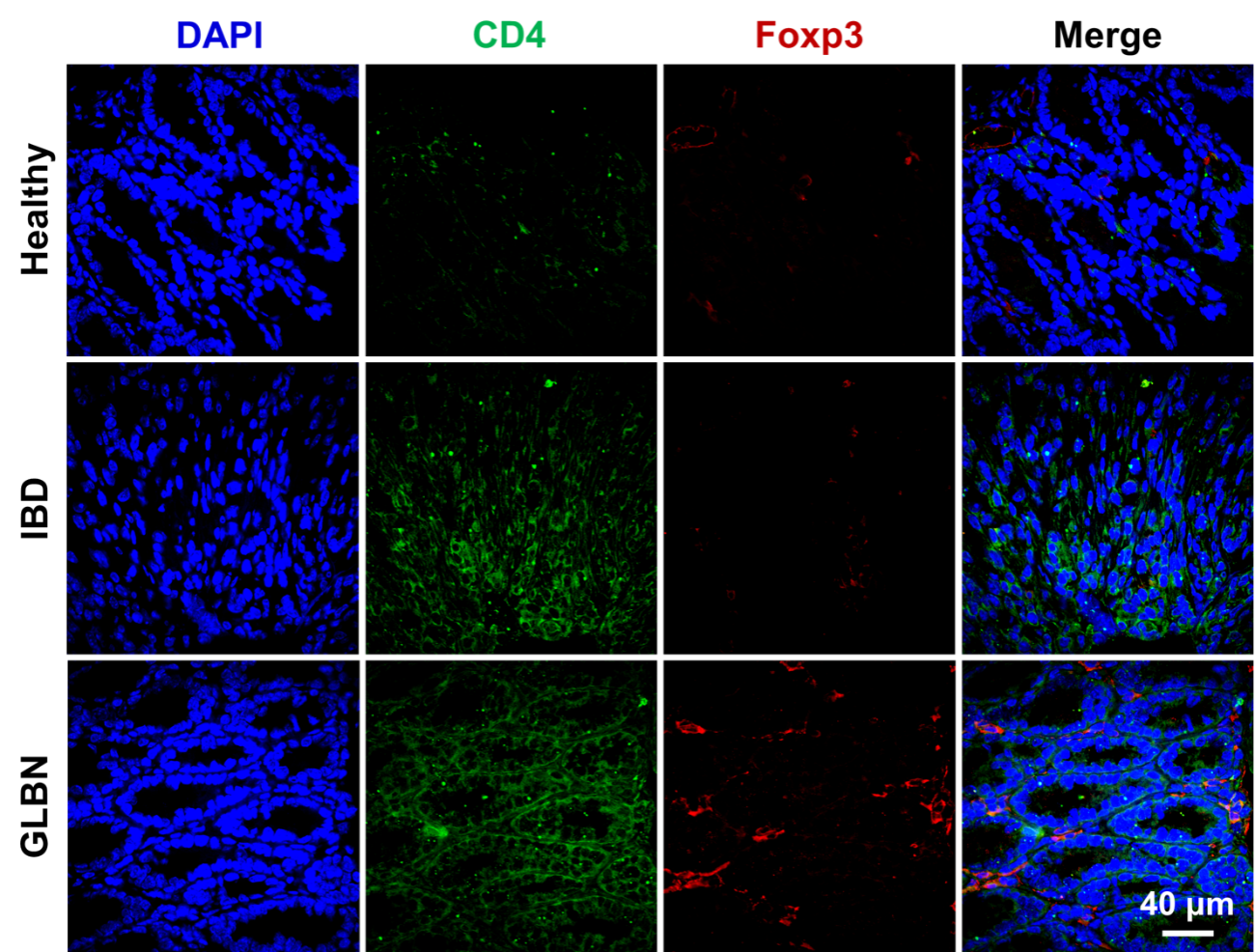


**Fig. S20** Immunofluorescence staining of Treg cells (CD4+Foxp3+) in the colon tissues of each group.

**Fig. S21** Relative abundance of Muribaculaceae-unclassified. Data are shown as mean ± S.D. *p* values were calculated via one-way ANOVA test. **p* < 0.05.

**Fig. S22** Relative abundance of *Akkermansia*. Data are shown as mean ± S.D. *p* values were calculated via one-way ANOVA test. **p* < 0.05.

| Gene | Forward primer | Reverse primer |
| --- | --- | --- |
| *TNF-α* | GGTGCCTATGTCTCAGCCTCTT | GCCATAGAACTGATGAGAGGGAG |
| *iNOS* | CCAAGCCCTCACCTACTTCC | CTCTGAGGGCTGACACAAGG |
| *Arg-1* | CCAGAAGAATGGAAGAGTCAGTGT | GCAGATATGCAGGGAGTCACC |
| *IL-10* | GGTTGCCAAGCCTTATCGGAAATG | GCCGCATCCTGAGGGTCTTC |
| *IL-6* | TACCACTTCACAAGTCGGAGGC | CTGCAAGTGCATCATCGTTGTTC |
| *CD163* | AATCACATCATGGCACAGGTCACC | TCGTCGCTTCAGAGTCCACAGG |

**Table S1** List of primers.
